# Ion Transport Enhancement in Charge-Regulating Hydrogels

**DOI:** 10.1021/acsnano.6c12908

**Published:** 2026-10-26

**Authors:** Pierre J. Walker, David Beyer, Christian Holm, Zhen-Gang Wang

**Affiliations:** † Division of Chemistry and Chemical Engineering, 6469 California Institute of Technology, Pasadena, California 91125, United States; ‡ Institute for Computational Physics, 9149 University of Stuttgart, D-70569 Stuttgart, Germany

**Keywords:** hydrogels, charge regulation, ion transport, acid−base reactions, molecular dynamics, Monte Carlo

## Abstract

Weak polyelectrolyte hydrogels are emerging as a versatile platform for ion transport and separation, desalination, and energy applications. However, a predictive understanding of how dynamic charge regulation at the nanoscopic scale influences ionic mobility in these polymer networks remains elusive. Here, using large-scale molecular simulations, we dissect the coupled mechanisms governing small-ion transport in weak polyelectrolyte hydrogels, and reveal how charge density, competitive binding, and dynamic charge regulation jointly control ion mobility. By systematically isolating these effects, we uncover a pronounced nonmonotonic dependence of ion self-diffusion on hydrogel p*K*_a_ and salt concentration for multivalent ions. Strikingly, allowing the hydrogel backbone to dynamically regulate its charge eliminates this nonmonotonicity and leads to dramatic enhancements in ion mobility, with diffusion coefficients increasing by more than a factor of 2 for trivalent ions. We attribute this effect to a protonation-assisted hopping mechanism that continuously remodels the local electrostatic landscape, providing an additional molecular-scale pathway for ion unbinding beyond translational escape and accelerating ion-pair kinetics without affecting the fraction of free ions. Our results demonstrate that dynamic nanoscale charge regulation can decouple ion mobility from equilibrium binding strength, offering a powerful design lever unavailable in fixed-charge materials. Building upon these insights, we propose simple molecular design principles for next-generation ion-conducting hydrogels and suggest alternative strategies for nanostructured membranes, energy materials, and biologically inspired charge-adaptive soft matter.

## Graphical abstract

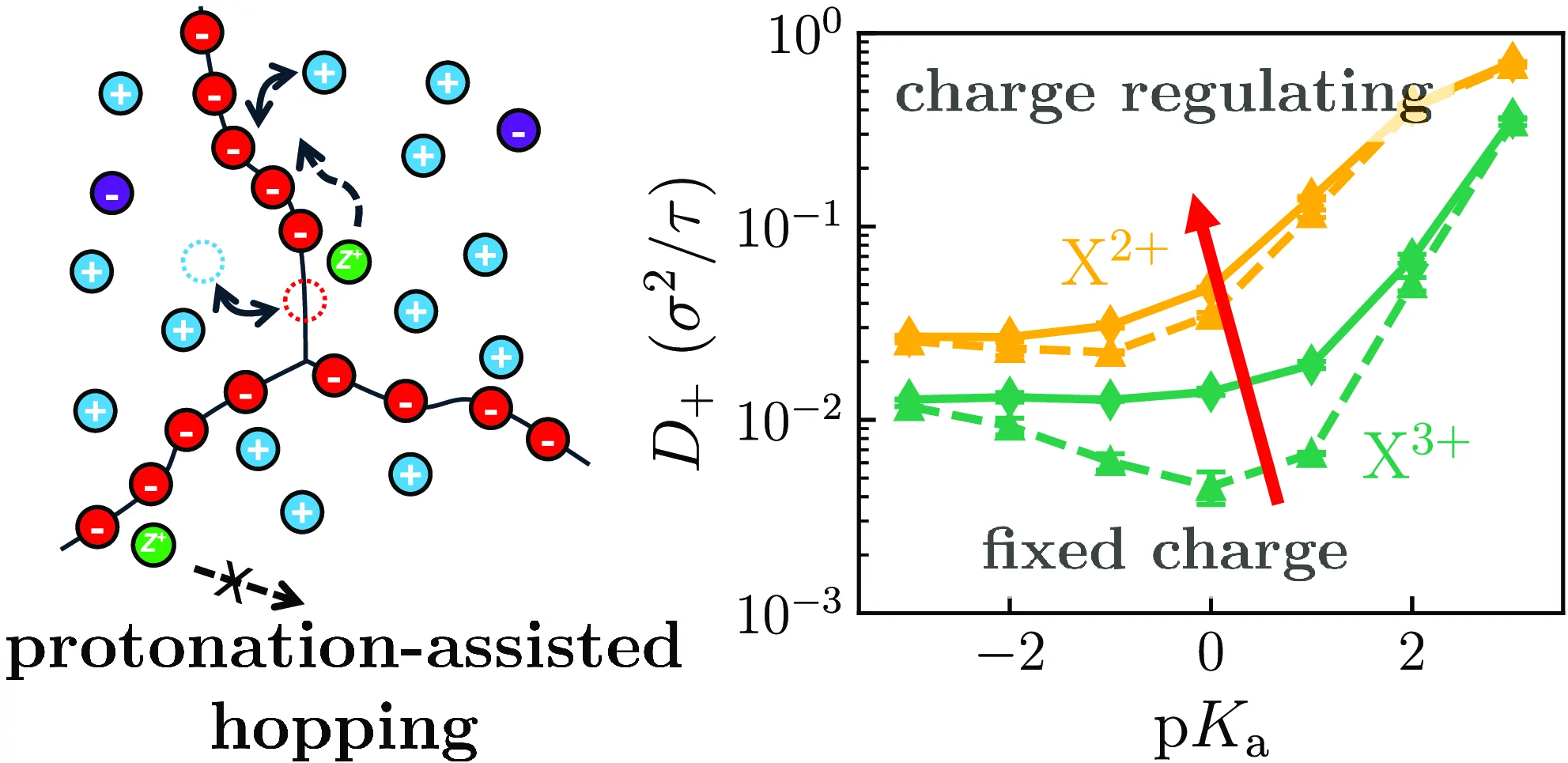

## 1 Introduction

Polyelectrolyte hydrogels—soft solids consisting of chemically or physically cross-linked polyelectrolyte chains—have emerged as a promising class of tunable materials with remarkable physicochemical properties.^1–6^ An important feature of these charged polymer networks is their exceptional swelling capacity in aqueous solutions, which is reactive to external stimuli, including salt,^7^ temperature,^5^ and light.^8^ This responsiveness gives rise to numerous advanced applications of polyelectrolyte hydrogels, ranging from desalination^6^ over electroadhesion^9^ and targeted drug delivery^10^ to nanomechanics.^11^

Although much of the experimental and theoretical focus has been on hydrogels composed of *strong* polyelectrolytes with a *fixed-charge* backbone, many hydrogels consist of *weak* polyelectrolytes, such as the commonly used poly­(acrylic acid).^12–14^ In contrast to strong polyelectrolytes, weak polyelectrolytes contain weakly acidic or basic groups, whose degree of ionization depends on the pH value.^15^ Naively, one might expect the behavior of weak polyelectrolytes to be equivalent to that of strong polyelectrolytes with the same fraction of ionized monomers. However, because the ionization state of these weak monomers is determined by reversible acid–base equilibria, weak polyelectrolytes often dynamically adapt their ionization state in response to various physicochemical factors, including system pH, salt concentration and valency, and even electrostatic interactions with other polyelectrolytes. Such *charge regulation* effects are known to play an important role in numerous systems in soft matter science and biophysics, for example weak polyelectrolyte brushes^16–22^ and complexes,^23–25^ globular^26–28^ and intrinsically disordered^29–31^ proteins, and nanoparticles^32–34^ and colloids.^35,36^ In weak polyelectrolyte hydrogels, charge regulation results in a pH-responsive swelling response,^37^ nontrivial interactions with client molecules^38,39^ and, in special cases, even a first-order swelling transition.^11,40,41^

The pH-responsive behavior of charge-regulating polyelectrolyte hydrogels has been studied in detail using experiments,^14,40^ (semi)­analytical theory,^41–43^ and coarse-grained simulations.^37,44–46^ However, virtually all existing theoretical studies on these systems have either focused on static equilibrium properties or large-scale conformational motions.^47,48^ In contrast, a mechanistic understanding regarding the transport of small (multivalent) ions and charged client molecules through charge-regulating polyelectrolyte networks has not been attained yet, although it is highly relevant to applications ranging from hydrogel-based membranes and filters to targeted drug delivery and the transport of molecules within biomolecular condensates. This lack of understanding is unsurprising, since charge regulation is inherently entangled with numerous other factors contributing to transport properties, including the salt concentration, ion binding, polymer density, and network architecture, all of which complicate the theoretical analysis. For example, while it is known that varying the charge fraction of a hydrogel can affect the transport properties of added salt, the very addition of salt (particularly with a high valency ion) to a charge-regulating hydrogel can shift the ionization equilibrium through favorable electrostatic interactions, changing the ionization fraction of the hydrogel in turn. Similarly, ionization equilibria, salt partitioning, and hydrogel swelling are all tightly coupled,^37^ which in turn influences the transport of small ions.

Despite the complexities described above, developing a molecular-level understanding of ion transport in charge-regulating hydrogels—linking nanoscopic phenomena with macroscopic bulk properties—is of paramount importance for the rational design of hydrogel-based materials across various applications and scientific disciplines. Gaining this understanding purely based on experimental observations is practically infeasible due to the challenges in disentangling the various factors explained above. It is therefore necessary to leverage theory and computer simulations to understand the various effects influencing ion transport in charge-regulating polymer networks. Whereas analytical approaches typically neglect many details that may be relevant for these complex systems and employ drastic approximations, molecular simulations at the all-atom level are computationally too expensive for studying large, charge-regulating hydrogels. The best compromise between these two extremes is to employ coarse-grained, implicit solvent simulations, which omit chemical specificity but maintain a particle-based description and fully account for nontrivial effects such as electrostatic correlations, chain connectivity, etc. It is for this reason that we leverage coarse-grained molecular dynamics (MD) simulations, in combination with the Reaction Ensemble Monte Carlo method^49,50^ (RxMC) in this publication: by reducing the system complexity to a few key design parameters, it is substantially easier to examine the contribution of isolated effects on macroscopic properties. To achieve this, we adopt a hierarchical strategy. Representing our polyelectrolyte hydrogel as a unit cell of a diamond-like network,^37,45,46,51,52^ as shown in Figure 1a, we progressively add layers of complexity (salt, charge regulation) step-by-step (Figure 1b). Further details regarding the simulation methods are given in section 4. As we show below, this approach allows us to obtain simple molecular design principles for next-generation ion-conducting hydrogels. These insights are subsequently leveraged to suggest new strategies for membranes, energy materials, and biologically inspired charge-adaptive soft matter.

**Figure 1:**
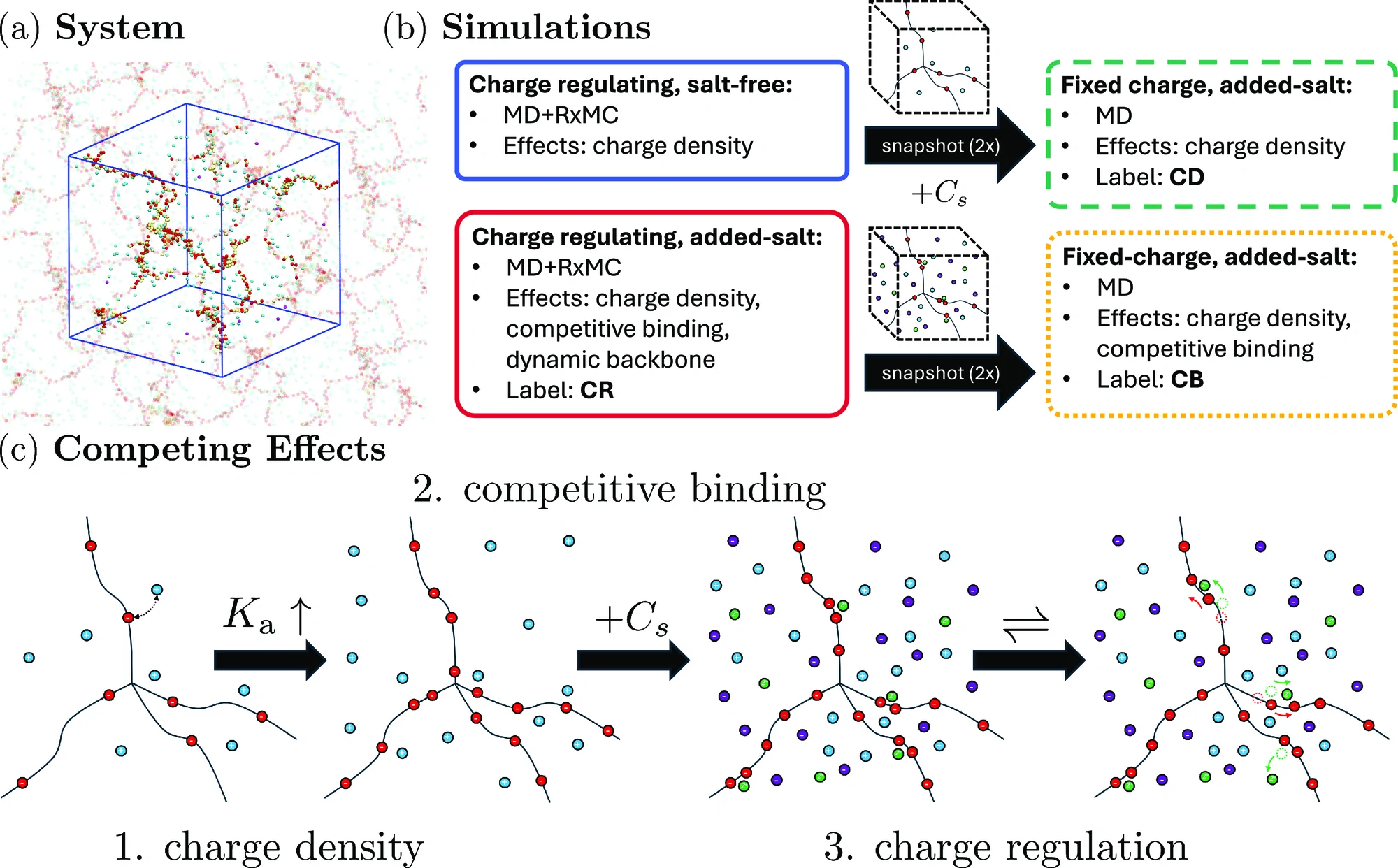
Methodology (a) Simulated hydrogel architecture using a diamond-like unit cell. (b) Summary of the types of simulations performed in this work, the approach used, and their respective labels. (c) Different competing effects investigated in this work: 1. variation in hydrogel charge density, 2. added-salt induced competitive binding, and 3. dynamical charge regulation of the hydrogel backbone.

## 2 Results and Discussion

In total, more than 700 distinct systems were examined in the course of this work. Presenting results for every case in the main text would be impractical; all data are therefore provided in the Supporting Information. The results shown here are selected to illustrate and elucidate the physical mechanisms of interest. The conclusions drawn are supported not only by the specific examples discussed below, but by the full ensemble of systems considered.

The remainder of this section is organized around systems of increasing complexity, designed to isolate the individual effects highlighted in Figure 1c.

### 2.1 Charge Density

From ideal-solution theory it can be derived that increasing the p*K*_a_ of the deprotonation reaction reduces the equilibrium degree of ionization α of the hydrogel according to

1 $$10^{-pK_{a}}=\left(\frac{C_{m}}{C_{0}}\right)\frac{\alpha^{2}}{1-\alpha }$$

Here, *C*_0_ = 1 M is the reference concentration and *C*_m_ is the concentration of ionizable monomers. In polymer networks, connectivity between neighboring beads renders adjacent ionized beads energetically unfavorable due to strong electrostatic repulsion. As shown in Figure 2a for charge-regulating hydrogels in the absence of salt, this repulsion suppresses ionization of the hydrogel backbone and gives rise to an apparent shift in the p*K*_a_. This well-known phenomenon in weak polyelectrolytes–illustrated in Figure 2a–is termed the polyelectrolyte effect and has been observed in several studies.^37,53,54^

**Figure 2:**
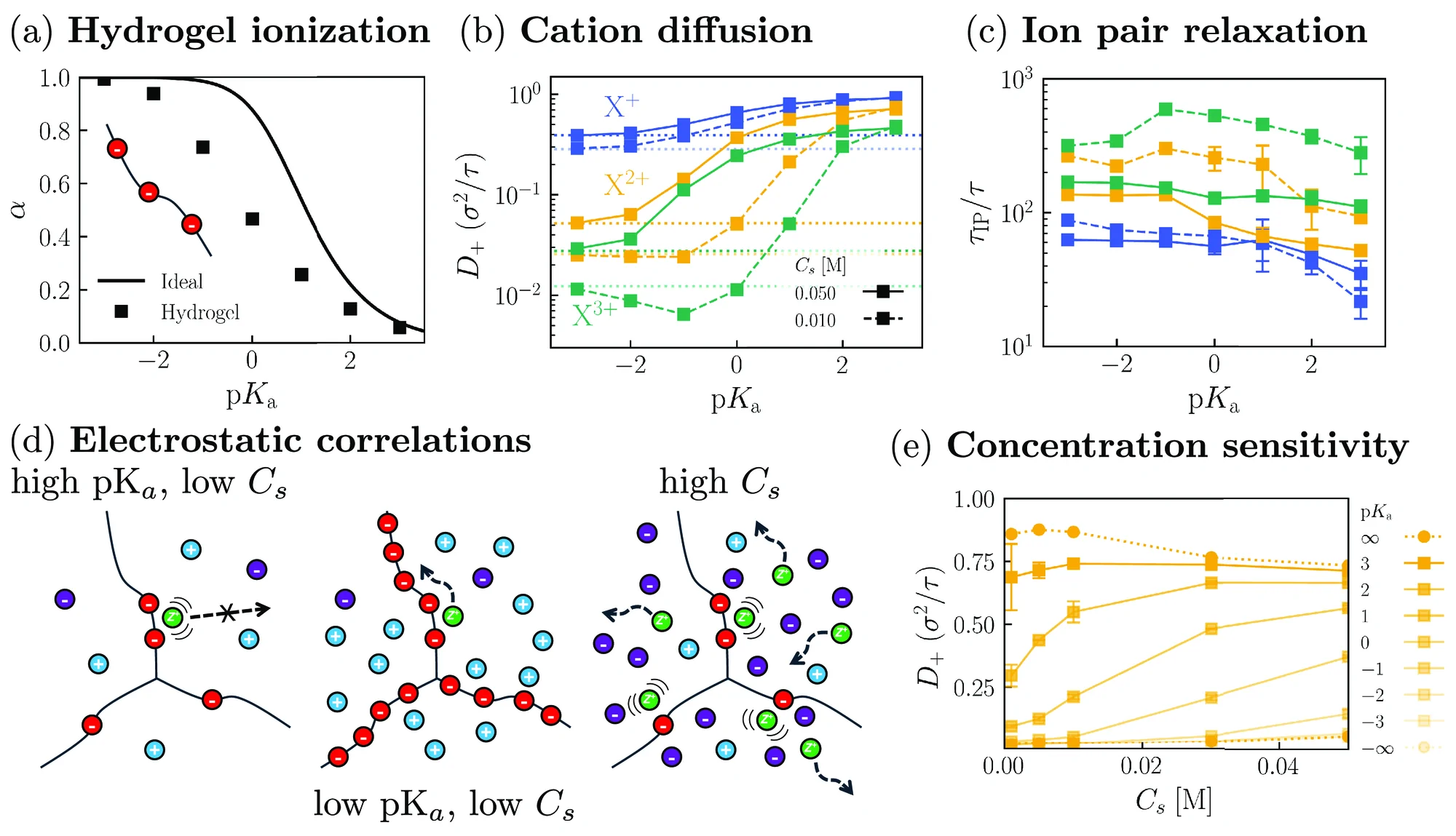
Charge Density (a) Variation in the hydrogel degree of ionization with varying p*K*_a_ in the absence of salt. The prediction for the ideal limit is shown as a black line. A schematic representation of the typical charge distribution along linear segments in CD simulations is also included. (b) Cation self-diffusion coefficient obtained using the CD simulations with varying p*K*_a_, cation valency and salt concentration. Colors denote valency (blue: monovalent, yellow: divalent, green: trivalent) and linestyles denote concentration (solid: 0.05 M, dashed: 0.01 M). Dotted lines denote the diffusion coefficient for a fully ionized hydrogel. (c) Ion pair relaxation times between the added cation and hydrogel backbone for the same conditions as (b), using the same colors and linestyles. (d) Schematic illustration of the effects of electrostatic correlations on the binding between the cation and the hydrogel backbone. (e) Variations in the salt-concentration dependence of *D*_+_ with varying p*K*_a_ for a divalent cation.

To capture realistic charge states of hydrogels at a given p*K*_a_ while isolating transport mechanisms, we first perform calculations by extracting equilibrated charge configurations from the charge-density simulations and quenching the backbone charges. Multivalent salts of varying concentration are then introduced, producing systems in which the hydrogel charge profile is fixed while the particle positions of monomers and mobile ions evolve dynamically. This process is illustrated in Figure 1b. These simulations allow the effect of the hydrogel charge density (labeled as CD) on cation transport to be examined independently of competitive binding and dynamical charge regulation.

The resulting self-diffusion coefficients as a function of cation valency, salt concentration, and p*K*_a_ are shown in Figure 2b. Overall, the self-diffusion coefficient decreases with increasing cation valency and increasing degree of ionization (decreasing p*K*_a_), while higher salt concentrations partially restore mobility. For high-valency cations at low salt and low p*K*_a_, a pronounced minimum in the diffusion coefficient is visible. In general, the effects of varying charge density are not surprising: if we consider small ions bound to the polymer backbone as practically motionless, only the fraction of unbound ions contributes to the self-diffusion coefficients. Increasing the degree of ionization (lowering the p*K*_a_) provides more sites to which the added cations can bind, thus reducing the self-diffusion coefficient. A similar effect occurs when increasing the ion valency. With stronger electrostatic interactions, the fraction of bound cations will increase, leading to a decreasing self-diffusion coefficient. Adding more salt (and thus cations) simply increases the amount of free ions, resulting in a larger self-diffusion coefficient. At sufficiently high ion concentrations, where electrostatic correlations and packing effects dominate, the self-diffusion coefficient would be expected to eventually decrease again.

Generally speaking, we find a good correlation between the fraction of free cations and the self-diffusion coefficient (see ESI Figure S8). However, for high valency cations, we observe a minimum in the self-diffusion coefficient at low salt concentrations and low p*K*_a_, which is not present in the unbound fraction. To explain this unexpected result, consider that in these regimes the fraction of free ions is extremely small. Therefore, the ions are bound most of the time and their effective diffusion coefficient is primarily governed by the rate of unbinding from the backbone rather than the diffusion coefficient of a free ion. Thus, the self-diffusion coefficient is determined by the lifetime of ion-polymer associations and should (approximately) be inversely related to the ion-pair relaxation time.^55,56^ This relationship is borne out in Figure 2c: divalent and trivalent systems exhibit relaxation times that decrease strongly with increasing salt concentration, consistent with enhanced electrostatic screening that weakens ion-polymer correlations and facilitates transport. At salt concentrations below 0.01 M, the ion-pair relaxation time for both multivalent ions displays a pronounced maximum at intermediate p*K*_a_, coinciding with the minimum in the self-diffusion coefficient. We attribute this nonmonotonic behavior to the change in binding environment for the added cations at low salt concentrations: as shown in Figure 2d, at high p*K*_a_ and low salt concentrations, the cation is strongly bound to the backbone, with its motion being coupled to the much slower hydrogel backbone. With decreasing p*K*_a_, more charged sites become available, such that it is favorable for the cation to translate along the backbone, increasing its diffusion coefficient. Distributing the charge uniformly along the hydrogel backbone eliminates the minima by providing easier mobility along the backbone (see ESI Figure S9). At very high salt concentrations, when the amount of cations exceeds the amount of available sites at all p*K*_a_, this minimum no longer appears as there are always free cations even when the hydrogel is fully charged. Although previous studies have explored the influence of charge fraction on ionic transport in hydrogels,^5,7,57,58^ to our knowledge such pronounced nonmonotonic behavior has not been reported.

Ion concentration provides an additional means of tuning transport, as shown in Figure 2e. We include the limiting cases of neutral (p*K*_a_ → ∞) and fully charged (p*K*_a_ → – ∞) hydrogels. In the neutral limit, self-diffusion decreases monotonically with concentration, as expected from crowding and electrostatic correlations in bulk electrolytes. Once the hydrogel carries charge, however, electrostatic binding strongly suppresses mobility at low salt concentrations. Increasing salt initially enhances diffusion by saturating polymer binding sites; at still higher concentrations, diffusion is expected to decrease again as ion–ion correlations and packing effects become dominant.

At intermediate p*K*_a_, where the hydrogel is only partially ionized, particularly for divalent and trivalent ions, the concentration dependence becomes highly sensitive to salt concentration. The strongest sensitivity to salt concentration occurs near p*K*_a_ ≈ 0, while either increasing or decreasing p*K*_a_ diminishes this effect. This regime coincides with the inflection points in the p*K*_a_-dependent trends of Figure 2b, corresponding to the maximum of the “charge regulation capacitance,”^26^ where the system is particularly sensitive to changes in p*K*_a_. Previous studies such as Yin et al.^7^ would likely have observed similar features had a broader range of charge fractions been examined.

Taken together, the results presented in this section underscore the delicate interplay between hydrogel charge density and salt content in controlling ion transport. As we will see in the following sections, the inclusion of additional physical effects causes these two seemingly independent parameters to become coupled.

### 2.2 Competitive Binding

The results discussed in the previous section are directly relevant to hydrogels synthesized from mixtures of fixed-charged and uncharged monomers. Examples of such fixed-charged groups include sulfonates, quaternary ammonium groups, and certain ionic-liquid-based monomers,^7^ which all have very small p*K*_a_ values. However, many commonly used monomers like carboxylic acids and phosphates exhibit more moderate p*K*_a_ values.^4,5^ In such systems, the deprotonation equilibrium can be strongly influenced by changes in solution conditions, for example by the addition of multivalent salts.^15^ As shown in Figure 3a, added salt shifts the equilibrium toward the deprotonated state due to favorable electrostatic interactions between cations and the polymer backbone, resulting in increased ionization fractions. While monovalent salts induce only minor changes, divalent and especially trivalent ions drive substantial shifts, with the latter pushing the degree of ionization beyond the ideal-solution limit at high concentrations, effectively reversing the polyelectrolyte effect.

**Figure 3:**
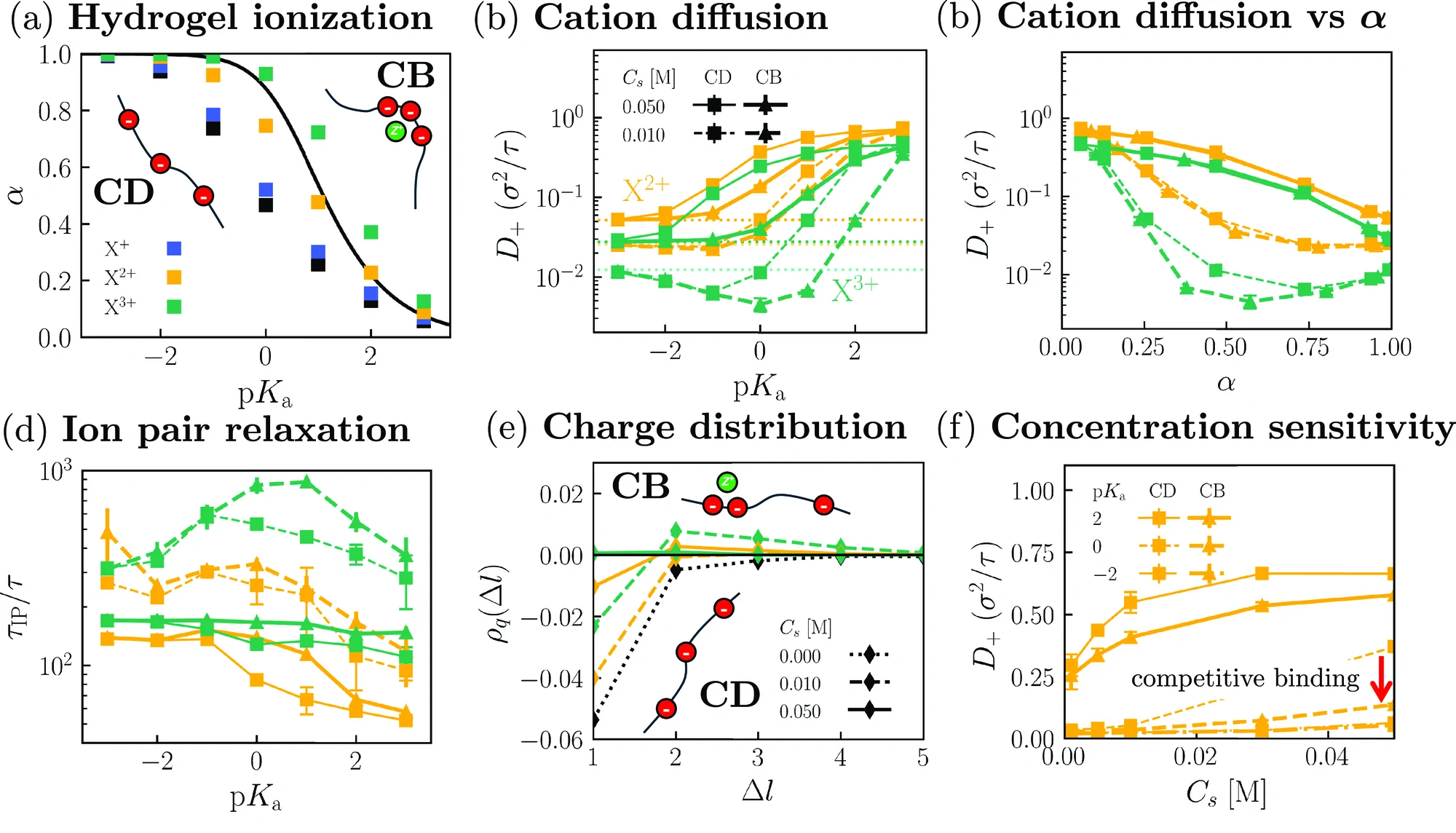
Competitive Binding (a) Variation in the hydrogel degree of ionization with varying p*K*_a_ in the presence of added salt (*C*_s_ = 0.05 M). Predictions from the ideal limit have been included. A schematic representation of the difference in charge distribution between CD and CB simulations along linear segments is also included. (b, c): Cation self-diffusion coefficient obtained using the CD (thin) and CB (thick) simulations with varying p*K*_a_ (b) and degree of ionization (c), cation valency and salt concentration. Colors denote valency (yellow: divalent, green: trivalent) and lines denote concentration (solid: 0.05 M, dashed: 0.01 M). Dotted lines denote the diffusion coefficient for fully ionized hydrogels. (d) Ion pair relaxation times between the added cation and hydrogel backbone for the same conditions as (b), using the same formatting scheme. (e) Charge profile autocorrelation with varying monomer separation Δl using the same scheme as (b) for p*K*_a_ = −1. (f) Variations in the salt-concentration dependency of *D*_+_ with varying p*K*_a_ for a divalent cation between CD and CB simulations..

To isolate the consequences of this salt-induced shift in ionization—without introducing dynamical charge regulation—we sample equilibrated charge profiles from charge-regulating simulations in the presence of added salt, quench the backbone charges, and perform additional competitive-binding (CB) simulations. This process is illustrated in Figure 1b. Because monovalent salts produce negligible changes in the ionization state, they do not lead to appreciable differences relative to the CD results discussed earlier. Accordingly, the remainder of this section focuses on divalent and trivalent ions.

The resulting self-diffusion coefficients are shown in Figure 3b. Qualitatively, the dependence on p*K*_a_ is identical to that observed in the CD simulations, reflecting the same competition between electrostatic binding and screening. There are, however, two quantitative differences. First, the inflection points in the self-diffusion curves shift between the two cases. This behavior mirrors the corresponding changes in ionization fraction shown in Figure 3a and in the fraction of unbound ions reported in the ESI, becoming more pronounced at higher salt concentrations, consistent with the stronger forward shift of the deprotonation equilibrium. A second, more unexpected difference is the appearance of deeper minima in the self-diffusion coefficient at low salt concentrations. Because the fraction of unbound ions is nearly identical between the CD and CB simulations (see ESI Figure S4), one might anticipate only a horizontal shift in the diffusion curves with p*K*_a_. This is particularly evident when one examines the dependence of the self-diffusion coefficient on the degree of ionization (Figure 3c): for the same degree of ionization, the self-diffusion coefficients from the CB simulations are consistently less than those from the CD simulations. The additional suppression of mobility for the same degree of ionization therefore points to a structural reorganization of the backbone charge profile that enhances the ion-pair lifetimes, suppressing cation diffusion. This interpretation is supported by the ion-pair relaxation times shown in Figure 3d, which exhibit correspondingly larger maxima at low salt concentrations. To probe the origin of this effect, we examine the correlation function of the charge profile along the polymer backbone (eq 8).

Representative results are shown in Figure 3e. In the absence of salt, the nearest-neighbor value of ρ_*q*_ is negative. This indicates that if a bead *i* is charged, its immediate neighbors preferentially remain neutral in order to minimize electrostatic repulsion, a direct manifestation of the polyelectrolyte effect. Upon the addition of multivalent salt, binding of the cations to the backbone locally reduces the effective negative charge density, making it more favorable for neighboring beads to deprotonate and thus increasing ρ_*q*_ at short separations. Notably, the largest increase occurs for next-nearest neighbors (Δl=2) . Such an alternating ionization pattern allows beads *i* and *i* + 2 to both carry charge while avoiding nearest-neighbor repulsion with bead *i* + 1. Furthermore, this pattern enables both sites to be bridged by a single multivalent cation.^59,60^ Examining $\rho_{q}\left(\Delta l=1\right)$ specifically, increasing salt concentration and cation valency progressively raises $\rho_{q}\left(\Delta l=1\right)$ from its salt-free value, ultimately rendering it positive for trivalent ions at *C*_s_ = 0.05 M. These results demonstrate that multivalent salts not only shift the overall degree of ionization, but also reorganize the spatial pattern of charges along the polymer backbone to maximize favorable ion-polymer interactions. This also manifests as an increase in the cation coordination number—the number of monomers electrostatically associated with a cation—at a given degree of ionization in the CB simulations relative to the CD simulations, as shown in ESI Figure S11. The resulting binding environments, illustrated in Figure 3a,e, hinder cation movement along the backbone, increasing ion-pair lifetimes and consequently further suppressing cation mobility relative to the CD case. Trends in $\rho_{q}\left(\Delta l=1\right)$ are examined over varying p*K*_a_, cation valency and added salt concentration within ESI Figure S6.

A practical consequence of salt-induced shifts in the deprotonation equilibrium is their impact on the concentration-dependence of ion transport. As discussed earlier, this effect is most pronounced at high salt concentrations, where forward shifts in ionization reduce the self-diffusion coefficient. Compared to systems with quenched charge profiles, this leads to a transport behavior that is more robust with respect to changes in salt concentration, as shown in Figure 3f. Although such behavior may be unfavorable for applications requiring highly responsive conductivity, it underscores the effect of charge sequence in tuning both thermodynamic and transport properties: even for a fixed average charge fraction, the local chemical environment can still be engineered to balance ion solubility and diffusion coefficient.

### 2.3 Dynamical Charge Regulation

Allowing backbone sites to dynamically regulate their charge state during the simulation is expected to further influence cation transport. The validity of extracting dynamical properties from simulations involving both RxMC moves and MD steps is addressed in Section 4.3. The results of charge-regulating (CR) simulations are shown in Figure 4a and compared with those of the charge-density (CD) and competitive-binding (CB) cases. We focus on a single salt concentration (0.01 M), both for clarity and because these effects are most pronounced at low concentrations where the fraction of unbound ions is small. Across all conditions examined, CR simulations consistently yield higher self-diffusion coefficients than CB systems with the same mean ionization fraction. At low p*K*_a_, the mobility of the cation can even exceed that observed in CD simulations with a lower charge density. The magnitude of this enhancement depends strongly on cation valency: divalent ions display only a small increase, whereas trivalent ions exhibit a more than 2-fold speed-up. This behavior is striking, given that CR simulations possess fractions of unbound ions that are comparable to those of the corresponding CB and CD systems. Moreover, for both valencies, the nonmonotonic minima observed previously in the self-diffusion coefficients disappear, demonstrating that dynamical charge regulation can overcome both electrostatic binding and the more favorable binding environments created by salt-induced charge reorganization.

**Figure 4:**
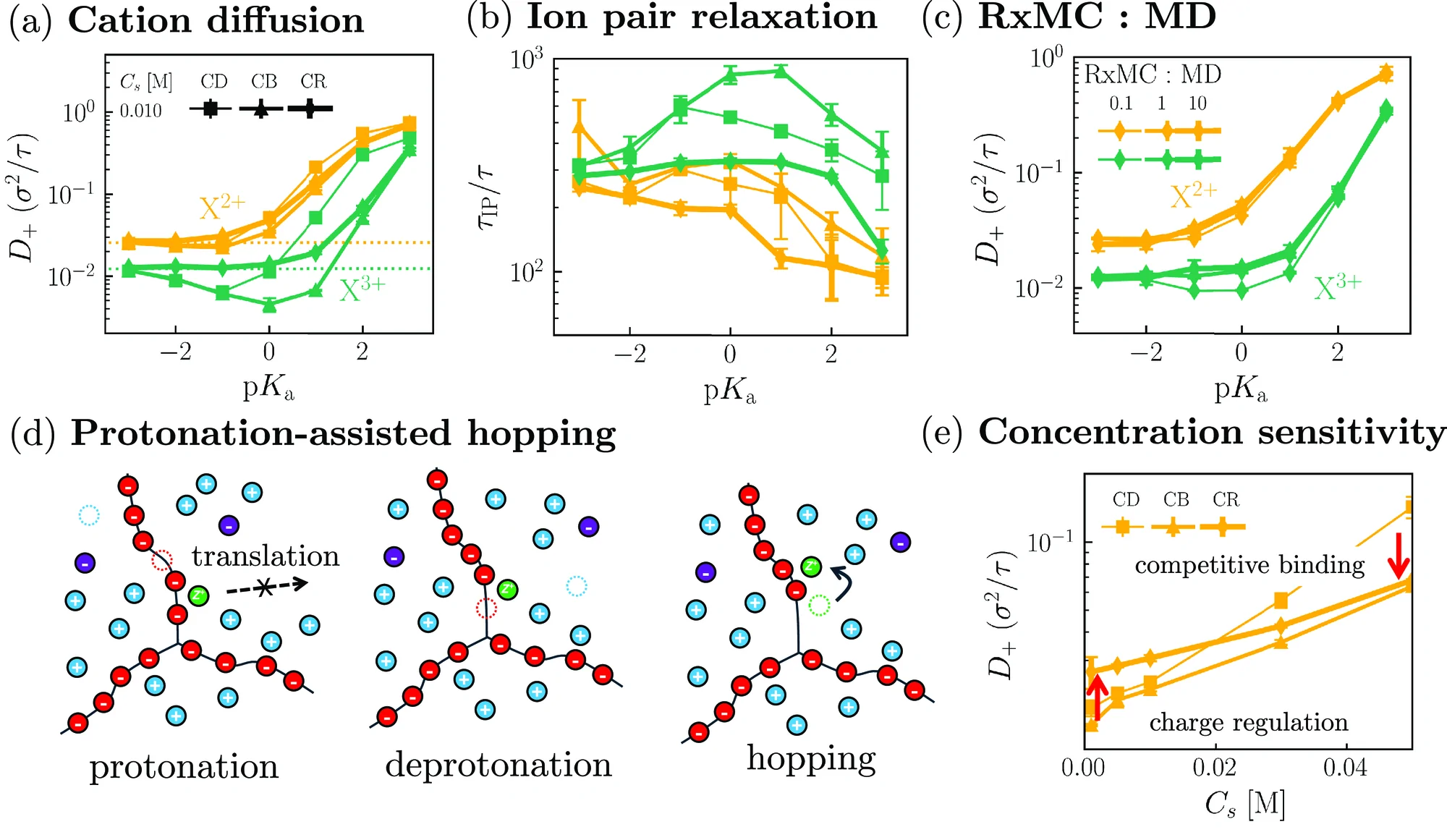
Charge Regulation (a) Cation self-diffusion coefficients obtained using the CR (thick), CB and CD (thin) simulations with varying p*K*_a_, cation valency and salt concentration. Colors denote valency (blue: monovalent, yellow: divalent, green: trivalent). Dotted lines denote the limited diffusion coefficient for a fully ionized hydrogel. (b) Ion pair relaxation times between the added cation and hydrogel backbone for the same conditions as (b), using the same colors. (c) Effect of varying the ratio of MC moves to MD steps on *D*_+_ obtained from CR simulations. (d) Visual illustration of the protonation-assisted hopping mechanism to enhance ion transport. (e) Variations in the salt-concentration dependence of *D*_+_ at p*K*_a_ = −1 for a divalent cation between the three simulation types.

The origin of the observed enhancement in diffusion can again be traced to the ion-pair relaxation times, shown in Figure 4b, which are systematically shorter in CR simulations than in their CB counterparts. As illustrated in Figure 4d, in CR systems, ion pairs can dissociate through two distinct mechanisms. The first mechanism is translational escape, whereby an ion diffuses away from the polymer backbone. As discussed earlier, this pathway strongly depends on the cation valency and the local binding environment. The second mechanism is unique to systems with dynamical charge regulation: the backbone site coordinating the ion may become protonated during an RxMC move. In general, the probability of such an event is governed by the p*K*_a_ value and the strength of the ion-polymer electrostatic interaction. Although protonation of a site is suppressed when a multivalent ion is bound to it, when protonation does occur, it is expected to be faster than translational escape. This additional pathway therefore shortens the ion-pair lifetime and increases the self-diffusion coefficient. Because binding is reversible, the equilibrium between bound and free ions satisfies

2 $$K_{\mathrm{bind}}=\frac{1-f_{\mathrm{free}}}{f_{\mathrm{free}}}\propto \frac{\tau_{\mathrm{IP}}}{\tau_{\mathrm{free}}}$$

where *f*_free_ is the fraction of unbound cations and τ_free_ denotes the lifetime of a free ion. This expression assumes mass-action kinetics and therefore neglects higher-order ion–ion correlations. Although τ_free_ is difficult to extract directly because free states are typically short-lived, *f*_free_ is identical in the CR and CB simulations. Equation 2 therefore implies that the reduced ion-pair relaxation times in CR systems must be accompanied by correspondingly shorter free-state lifetimes.

In CR simulations, the concomitant shortening of both τ_IP_ and τ_free_ can be attributed to the constraints imposed by protonation moves: for a charged site to protonate with appreciable probability, a nearby monomer must be charged and unoccupied. Once protonation occurs, a multivalent cation can rapidly migrate to this neighboring site and rebind. This correlated, protonation-assisted hopping process circumvents the otherwise large energetic penalty associated with protonating a site directly coordinating a multivalent ion, thereby accelerating both binding and unbinding kinetics and enabling faster ion motion. The described mechanism is most effective at low p*K*_a_, where the binding of cations is substantial, yet a sufficient number of charged sites remain available. This behavior is almost similar to a hydrogel where the total charge has been uniformly distributed, shown in ESI Figure S9. It is also consistent with the finding that, in this p*K*_a_ region, the fraction of high-valency cations binding to different hydrogel strands is slightly lower in CR simulations compared to CB simulations, as the cation can now move along a strand (see ESI Figure S12). It is also loosely analogous to Grotthuss proton hopping in water.^7,61^

The rate of protonation events is controlled in part by the ratio of RxMC moves to MD steps. One would therefore expect that, in the limit of very infrequent RxMC moves, CR simulations should approach CB behavior. To test this expectation, we varied this ratio by 2 orders of magnitude (see Figures 4c and ESI S10). With increasing RxMC frequency, the ion-pair relaxation time decreases while the self-diffusion coefficient increases; however, these changes are far smaller than would be anticipated from the magnitude of the rate variation itself. This observation indicates that the protonation-assisted hopping mechanism is not strongly limited by the intrinsic protonation rate.

As noted earlier, dynamical charge regulation enhances cation mobility primarily at low salt concentrations, whereas competitive binding suppresses transport at higher concentrations. The combined effect is therefore a reduced sensitivity of the self-diffusion coefficient to salt concentration, as shown in Figure 4e. This impact is most pronounced near the p*K*_a_ values at which the minima previously occurred: for p*K*_a_ ≤ 0, the self-diffusion coefficient no longer increases monotonically with concentration and even exhibits weak nonmonotonicity for trivalent ions at low salt concentrations.

## 3 Conclusion and Outlook

In this work, we have deconstructed the physical mechanisms governing ion transport in charge-regulating hydrogels. Using coarse-grained simulations, we have shown that the self-diffusion coefficient of small ions is determined by a competition between three mutually coupled factors: (1) electrostatic binding, which impedes ion motion; (2) electrostatic screening, which facilitates transport; and (3) charge regulation itself, which can either suppress or enhance mobility depending on conditions. These effects are strongly coupled, making it challenging to rationally design charge-regulating materials. Additional effects not included in our model could further modulate the behavior of weak polyelectrolyte hydrogels. First, the coarse-grained model employed in this work was deliberately simplified: it lacks chemical specificity, enabling us to identify universal trends that transcend the details of any particular system. Real multivalent ions, however, typically exhibit strong ion-specific effects that strongly influence gel behavior.^62,63^ Even so, ion specificity is expected to primarily affect the binding strength of the multivalent ions to the hydrogel backbone, but not the qualitative conclusions derived from our simulations. Second, we note that in reality the equilibrium swelling degree and salt concentration of weak polyelectrolyte hydrogels are strongly coupled to the ionization state,^37^ whereas, in our model, both are imposed. A self-consistent treatment of ionization and swelling equilibria can be achieved using the grand-reaction method,^64^ but lies beyond the scope of the present study. Third, in many technological applications of charge-regulating hydrogels, ion transport is induced through an externally applied voltage, which in turn couples both to the ionization state and the gel swelling state,^65,66^ demanding a self-consistent treatment of acid–base reactions and gel swelling. The effects induced by an external electric field are not modeled in the present work, which probes ion dynamics through simulations at thermodynamic equilibrium. Nevertheless, even under an applied DC field, the system is expected to eventually relax to a nonequilibrium steady state, corresponding to a stationary state of the dynamical acid–base reactions. Provided the field is not too strong and does not substantially alter the ionization equilibrium, we expect our main qualitative conclusions regarding the role of acid–base equilibria to carry over to this case. Building upon the insights gained through our coarse-grained simulations, we can hence formulate simple design heuristics for charge-regulating materials, anticipating future developments in both experiments and simulations. Accordingly, we conclude by outlining several promising directions for future experimental and theoretical investigations.

One important application of polyelectrolyte hydrogels is in ion-selective membranes, illustrated schematically in Figure 5a. In this context, the relevant performance metric is the relative permeability,

3 $$P_{\mathrm{gel}}=D_{\mathrm{gel}}K_{\mathrm{gel}}$$

where *P*_*i*_, *D*_*i*_, and *K*_*i*_ denote the permeability, diffusion coefficient, and partition coefficient in medium *i*, respectively. Charge-regulating hydrogels offer a distinct advantage over fixed-charge systems: for a given mean charge fraction, ion solubilities are expected to be comparable. In contrast, the enhanced diffusion coefficients in charge-regulating networks should translate directly into higher permeability. As shown above, this enhancement is most pronounced for multivalent ions and can reach more than a factor of 2 depending on the hydrogel p*K*_a_. This feature is particularly relevant for heavy-metal separations, where target ions often possess a high valency. Going beyond point-like small ions, it could potentially also be relevant for charged oligomers such as peptides in various biochemical and pharmaceutical processes. We emphasize again that the present model treats ions in a simplified manner and neglects the polarizability and specific complexation effects that are known to be important for many heavy metals. Although it is not straightforward to predict how such chemical specificity would modify transport under charge regulation, we anticipate that protonation-assisted hopping should still provide a substantial kinetic advantage. Developing a more detailed understanding of specific systems will require either chemically specific coarse-grained models^67,68^ or even atomistic simulations in the future, in which charge regulation can be included using a range of different algorithms.^69–74^

**Figure 5:**
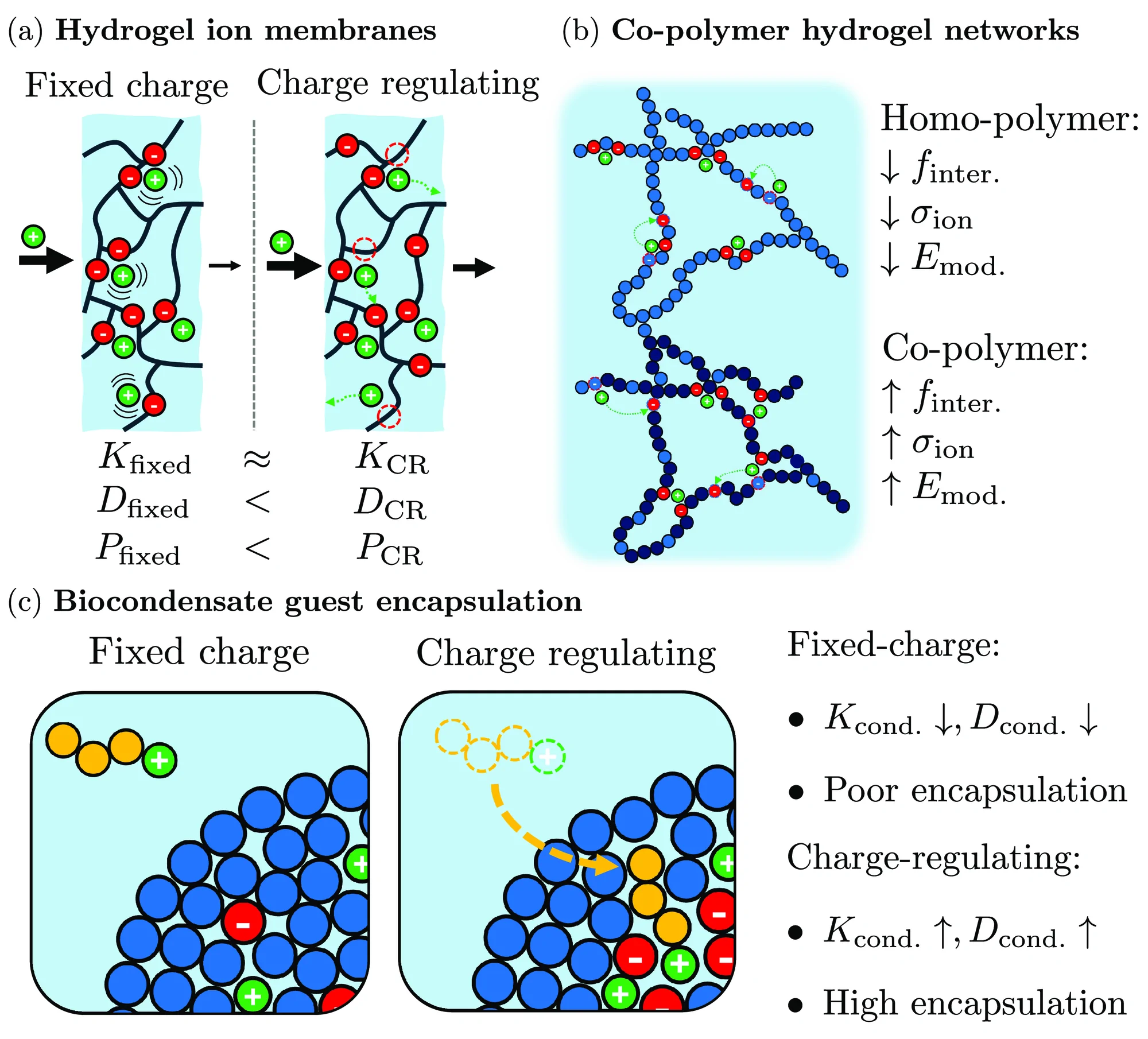
Hypothesized implications of charge regulation for ion transport. (a) Hydrogel ion membranes: we expect that for the same charge profile, a charge regulating hydrogel should have the same ion partitioning as the fixed charge equivalent, but with enhanced diffusion and, therefore, permeability. (b) Copolymer hydrogels: we propose that introducing neutral spacer monomers into a weak polyelectrolyte hydrogel should reduce the effects of competitive binding, increasing the fraction of interchain cross-links (*f*_inter_) and improving the ion transport (σ_ion_) and mechanical properties of the gel, such as the elastic modulus (*E*_mod._). (c) Biocondensate client encapsulation: we hypothesize that the adaptive environment of a charge-regulating biocondensate may improve the uptake of client molecules and enhance their diffusion coefficient.

A central limitation of charge regulation identified in this work arises from competitive binding: the hydrogel adapts its charge profile to maximize favorable ion-polymer interactions, thereby reducing mobility at high concentrations. In applications such as sensors or battery electrolytes, where maximizing ionic transport is essential, mitigating this effect is crucial. Furthermore, only cation bridges between different strands contribute to mechanical stability of the gels. As illustrated in ESI Figure S12, the conditions where the fraction of intercross-links is maximized depends on the ion valency, added salt concentration and p*K*_a_, all of which, in weak polyelectrolytes, become convoluted. A more tractable strategy is to employ copolymer networks in which charge-regulating monomers are interspersed with neutral units, as illustrated in Figure 5b. We hypothesize that neutral spacers would increase the average separation between charges, suppressing local overcharging and the associated long ion-pair lifetimes. In this regime, ion-mediated cross-links between polymer chains are expected to be preferable to ions being locally sequestered along a single backbone. Separating charge-regulating sites entropically favors interchain bridges,^75^ while the increased ionic mobility associated with charge regulation should further benefit mechanical response relative to fixed-charge networks. Determining the optimal spacing between ionizable groups, such that protonation-assisted hopping remains effective while local overcharging is avoided, represents an intriguing direction for future work.

Another important avenue concerns the temperature dependence of ion transport. Temperature affects thermal motion, electrostatic interactions through the Bjerrum length, and the acid–base equilibrium itself. Because p*K*_a_ values typically decrease with increasing temperature,^76–79^ our results suggest that heating could suppress ion mobility by increasing the degree of ionization, even as thermal agitation and reduced electrostatic coupling act in the opposite direction. Exploring this interplay may shed light on the unusual temperature sensitivity observed in some charge-regulating hydrogels, particularly when neutral spacers are incorporated.^5^

Beyond synthetic materials, the concepts developed in this work have potential relevance in biological contexts. Charge-regulating hydrogels have qualitative similarities to biomolecular condensates,^80^ in which many amino-acid residues titrate with pH. For charged client molecules entering such chemically distinct environments,^81–83^ charge regulation may simultaneously enhance solubility, by producing a more favorable local environment for the client, and accelerate transport, improving encapsulation efficiency (Figure 5c). In the present work, we considered only a charge-regulating background matrix, but many client molecules—such as small-molecule pharmaceutical compounds, flexible polypeptides, and globular proteins—also undergo protonation equilibria.^84^ When both matrix and solute can adapt their charge, interactions may become more favorable than in fixed-charge systems,^26,38,85,86^ and protonation-assisted hopping could be further amplified because charge fluctuations of either component may trigger unbinding events. These hypothesized effects have clear implications for applications like drug delivery and nucleic-acid–based therapeutics, and warrant further simulation studies.

Overall, by disentangling the coupled phenomena underlying charge regulation and ion transport, this work opens multiple avenues for future exploration across membrane science, energy materials, soft-matter mechanics, and biological physics. We anticipate that combining charge regulation with architectural control of polymer networks will provide a powerful framework for designing next-generation ion-conducting materials.

## 4 Methods

### 4.1 Model

Following the approach used in previous simulation studies,^37,45,46,51,52^ we represent our polyelectrolyte hydrogel as a unit cell of a diamond-like network, as shown in Figure 1a. In this architecture, polymer strands consisting of *N* = 60 beads connect tetravalent cross-linking nodes. Each polymer bead is assumed to be a weak acid (HA), which can dissociate into an anionic bead (A^–^) by releasing a free cation (H^+^) into solution:

4 $$\mathrm{HA}⇌A^{-}+H^{+}$$

This chemical reaction is governed by the equilibrium constant $K_{a}=\frac{a_{A^{-}}a_{H^{+}}}{a_{\mathrm{AH}}}$ (p*K*_a_ = −log_10_ *K*_a_), where a_*i*_ is the activity of species *i*.

In addition to polymer chains and counterions, a *Z*:1 electrolyte is added to the system at a concentration *C*_s_. The cations X^*Z*^, acting as counterions for the polyelectrolyte network, have valency *Z* (+1, + 2, or +3). The anions X^–^ are always monovalent.

HA, A^–^, H^+^, and X^–^ beads all have the same effective diameter σ = 0.355 nm; in a previous work, this value was shown to accurately reproduce the excess chemical potential of aqueous NaCl solutions.^64^ The cations X^*Z*^ are half the size of the other beads (σ = 0.1775 nm) to distinguish them from H^+^ and to account for the smaller solvation diameter of higher-valency ions.^87^ All particles have the same mass *m* = 1.0 in the system of reduced Lennard-Jones units. The solvent is taken to be water at *T* = 298.15 K and is treated implicitly, such that the Bjerrum length is λ_B_ = 0.71 nm. For a full description of the interaction potentials, we refer the reader to the ESI.^88–93^

To characterize the effects of charge regulation on small-ion transport, we vary three parameters: the valency of the added salt cation (*Z* = +1, +2, +3), the salt concentration, for which we consider five values (*C*_s_ ∈ {0.001, 0.005, 0.01, 0.03, 0.05} M), and the equilibrium constant of the acid–base reaction, spanning p*K*_a_ = −3 to 3. As most hydrogels are primarily comprised of water, we assume a high swelling ratio such that the polymer volume fraction is low (ϕ ≈ 0.05, corresponding to *C*_m_ ≈ 0.16 M). This monomer concentration lies in the lower regime of typical hydrogels and was chosen to minimize screening contributions from ions originating within the network itself, thereby amplifying changes induced by the addition of multivalent salts, which are the focus of this work.

In general, the equilibrium volume of a charge-regulating polyelectrolyte hydrogel is determined by a complex balance of acid–base equilibria, ion partitioning, electrostatic interactions, and network elasticity.^37^ As we will show in the following, even at fixed volume, ion transport in charge-regulating hydrogels involves a convolution of multiple factors that are difficult to isolate. Allowing the network to swell or contract would only further complicate this analysis. Therefore, in the present work, we fix the monomer concentration across all simulations in order to focus specifically on transport mechanisms. An extension of this work to account for gel swelling and volume changes represents a natural direction for future research.

### 4.2 Simulation Methods

In this work, we identify three key effects that influence ion transport in charge-regulating hydrogels (see Figure 1c): (1) the influence of hydrogel charge density on ion transport, (2) competitive binding of cations to the hydrogel backbone, and (3) the role of *dynamical* charge regulation. Performing simulations that account for all three effects simultaneously would make it difficult to disentangle their individual contributions. We therefore perform three types of simulation that progressively add complexity, allowing the role of each effect to be isolated. The setup of these different types of simulation is summarized in Figure 1b and will be explained in detail in the following paragraphs.

In all simulations, we used molecular dynamics (MD) in conjunction with a Langevin thermostat to simulate the time evolution of the bead positions at a fixed temperature. The stochastic Langevin equations are solved numerically using the Velocity-Verlet integrator with a time step of Δ*t* = 0.01 τ, where τ is the Lennard-Jones time scale. We set the bare friction coefficient to γ = 1.0 τ^–1^, corresponding to a diffusion coefficient of *D* = 1.0 σ^2^τ^–1^ for noninteracting beads. We have chosen not to explicitly account for the effects of long-range hydrodynamic interactions using methods such as DPD^94^ or Lattice-Boltzmann^95^ in order to fully isolate the effects of charge regulation. The simulations are conducted using version 4.2.4 of the open source simulation suite ESPResSo.^96,97^

#### 4.2.1 Salt-Free Simulations

We first perform simulations of the hydrogel in the absence of added salt. To emulate changes in the hydrogel charge density with decreasing p*K*_a_ (Figure 1c), we combine MD with the Reaction Ensemble Monte Carlo method^49,50^ (RxMC). The RxMC method is one of several Monte Carlo schemes that allow simulating acid–base equilibria in weak polyelectrolytes.^15,49,50,98,99^ In this work, we choose RxMC rather than the grand-reaction method,^15^ as the latter would additionally capture effects such as gel swelling^37^ and ion partitioning,^64^ which are intentionally excluded here to simplify the physical picture.

To reach the desired polymer volume fraction, we use a simulation protocol similar to previous works:^37,44^ after initializing the gel network in a fully extended state, the simulation box is compressed isotropically at a rate of 0.1 σ/τ until the target monomer concentration is reached. The system is then equilibrated for 10^5^ MD steps, during which 10^3^ RxMC moves are attempted every 10^3^ MD steps. This procedure allows the network to relax and the backbone charges to reach equilibrium. Subsequent production runs are performed for 2 × 10^7^ MD steps, maintaining the same ratio of the MD steps to the MC moves. This ratio is consistent with previous simulations studying the effects of dynamical charge regulation^11^ and is intended to emulate the rapid kinetics of typical acid–base reactions,^100–102^ assisted by Grotthuss proton hopping.^61^

Although the salt-free simulations do not provide information on the transport of multivalent ions, they characterize the ionization equilibrium and charge distribution within the gel network. Because the simulations are performed in the absence of multivalent salt, they allow the influence of hydrogel charge density to be examined independently of competitive binding. To study ion dynamics in a hydrogel with a given charge density, we randomly select equilibrated charge configurations from the RxMC trajectories (including monovalent counterions), freeze the backbone charges, and add multivalent salt at varying concentrations. These systems are then simulated for an additional 2 × 10^7^ MD steps without RxMC. In this way, we isolate the effect of hydrogel charge density on ion transport. The described procedure is repeated for two independent charge realizations in order to average over charge fluctuations of the gel network. We refer to these quenched-charge simulations as charge-density (CD) simulations, after the effect being probed in these simulations (see Figure 1b).

#### 4.2.2 Added Salt Simulations

Having isolated the effect of charge density, we next examine the combined influence of competitive binding and charge regulation. These effects are captured in simulations performed with MD+RxMC in the presence of added salt. We therefore initialize the gel network with multivalent salt and repeat the protocol described above: isotropic compression at a rate of 0.1 σ/τ, equilibration for 10^5^ MD steps, and production runs consisting of 2 × 10^7^ MD steps, with 10^3^ RxMC moves attempted every 10^3^ MD steps. These simulations are referred to as charge-regulating (CR) simulations. From the production trajectories, we extract self-diffusion coefficients of the added salt ions; the validity of this procedure is discussed below. We also examine the effect of varying the ratio of MD steps to MC moves in the Discussion and Supporting Information.

In the CR simulations, the effects of charge regulation and competitive binding are intrinsically coupled. To separate these contributions, we again select two equilibrated gel charge configurations from the CR trajectories, freeze the backbone charges, and perform additional simulations for 2 × 10^7^ MD steps without RxMC. Because the mean degree of ionization and charge distribution are preserved, these simulations remove dynamical charge regulation while retaining competitive binding. These systems are referred to as competitive-binding (CB) simulations (see Figure 1b).

### 4.3 Analysis

All analysis in this work is performed using the programs MDAnalysis^103^ and MDCraft.^104^ Error bars for each property were obtained through block averaging of the trajectories. For CD and CB simulations, which sample two charge realizations of the hydrogels, block averaging is carried out across both trajectories. These have been included in all figures within the main text.

#### 4.3.1 Self-Diffusion Coefficients

The primary quantity of interest is the self-diffusion coefficient of the added ions, defined as

5 $$D_{i}=\lim_{t\to \infty }\frac{1}{6tN_{i}}\sum_{j=1}^{N_{i}}\left\langle \right|R_{j}\;\left(t\right)-R_{j}\;\left(0\right){|}^{2}\rangle$$

where *t* is time, *N*_*i*_ is the number of particles of species *i*, and **R**_*j*_(*t*) is the position of particle *j* at time *t*.

For simulations that include Monte Carlo steps (CR simulations), the validity of measured dynamical properties such as self-diffusion coefficients warrants careful consideration. In this work, transport properties are extracted exclusively for species that do not participate directly in Monte Carlo moves, such that their microscopic motion is governed solely by molecular dynamics. Consequently, we do not report the total ionic conductivity of the system, which would additionally require tracking the dynamics of the charged polymer backbone and protons, both of which are involved in RxMC moves.

Because particle motion is correlated, unphysical displacements associated with RxMC moves could in principle indirectly affect the dynamics of all species. Under the dilute conditions considered here, however, the influence of low-valency particles undergoing RxMC moves is expected to enter primarily through their collective contribution to electrostatic screening. When averaged over long trajectories, their effect is therefore anticipated to be comparable to that of physically diffusing particles. To validate this assumption, we explicitly examined the effect of varying the ratio of RxMC moves to MD steps over 2 orders of magnitude. These tests, reported in the Supporting Information, indicate that while small deviations arise in low-salt, low-p*K*_a_ regimes, for most conditions the measured dynamical properties are not significantly affected by this ratio.

#### 4.3.2 Ion Pairing

To assess how charge regulation influences the transport of multivalent ions, we quantify the fraction of cations not bound to the anionic sites on the hydrogel backbone. An ion is defined as unbound when no anions are present within a distance 1.5σ of the cation center (corresponding approximately to the location of the first minimum in the radial distribution function). Because the fraction of bound cations is an equilibrium quantity, its averaged value is independent of the ratio of MD to RxMC steps; a small ensemble-dependent finite-size effect may in principle exist, but is not expected to be significant here.

Although equilibrium properties are expected to be identical between CR and CB simulations up to finite-size effects, their relaxation dynamics may differ. Of particular relevance is the ion-pair relaxation time,^55,56,105^ which has been shown to correlate strongly with ionic self-diffusion. Following previous work,^105^ we define the ion-pair relaxation time τ_IP_ as the time at which the survival probability *P*(*t*) decays to 1/*e*. This quantity is obtained from the autocorrelation function of *h*_*ij*_(*t*)­

6 $$P\left(t\right)=\langle h_{\mathrm{ij}}\;\left(t\right)h_{\mathrm{ij}}\;\left(0\right)\rangle_{\mathrm{ij}}-\langle h_{\mathrm{ij}}\;\left(t\right)\rangle_{\mathrm{ij}}^{2}$$

where ⟨·⟩_*ij*_ denotes averages over all added cations and anionic hydrogel beads, and

7 $$h_{\mathrm{ij}}\;\left(t\right)=\{\begin{array}{ll} 1, & ∥R_{i}\;\left(t\right)-R_{j}\;\left(t\right)∥\leq 1.5\sigma_{\mathrm{ij}}\\ 0, & ∥R_{i}\;\left(t\right)-R_{j}\;\left(t\right)∥>1.5\sigma_{\mathrm{ij}}. \end{array}$$

Because the ion-pair relaxation time is a dynamical property, its validity in the presence of Monte Carlo steps has to be justified using arguments analogous to those applied to the self-diffusion coefficients. Across simulations in which the ratio of RxMC moves to MD steps was varied by 2 orders of magnitude, τ_IP_ was found to change at most by a factor of 2.

#### 4.3.3 Charge Profile Autocorrelation Function

The addition of high-valency ions through competitive binding is expected to modify the charge distribution along the hydrogel backbone. To quantify these changes, we define the autocorrelation function of the charge profile,^106^ averaged along the polymer strands of the hydrogel (disregarding cross-linkers)

8 $$\rho_{q}\;\left(\Delta l\right)=\langle q_{l}q_{l+\Delta l}\rangle_{\mathrm{chain}}-\langle q\rangle_{\mathrm{chain}}^{2}$$

Here, $q_{l}$ is the (dimensionless) charge of bead l and ⟨·⟩_chain_ denotes averages along the polymer strands. The quantity ⟨*q*⟩_chain_ is equivalent to the degree of ionization α. $\rho_{q}\left(\Delta l\right)$ is positive when beads separated by Δl along the chain are, on average, more highly charged than the bulk average, and negative in the opposite case.

## Supplementary Material



## Notes

Details regarding the simulation model and method are provided within the Supporting Information; additional figures containing all simulation results (degree of ionization, diffusion coefficient of cation and anion, ion-pair relaxation time, fraction of free ions, charge profile autocorrelation function at Δl=1 ) are also included. A preprint version of this manuscript is available: Pierre J. Walker, David Beyer, Christian Holm, and Zhen-Gang Wang; ion Transport Enhancement in Charge-Regulating Hydrogels; 2026 10.26434/chemrxiv.15004897/v2; ChemRxiv. https://chemrxiv.org/doi/abs/10.26434/chemrxiv.15004897/v2 (accessed October 8, 2026) (PDF)

## ∥

Department of Theoretical and Computational Biophysics, Max Planck Institute for Multidisciplinary Sciences, D-37077 Göttingen, Germany
