# Supplementary material for: Ion Transport Enhancement in Charge-Regulating Hydrogels
Source: ACS Nano. 2026 Oct 26;20(44):29889–901. doi: 10.1021/acsnano.6c12908 (PMC13758397; doi:10.1021/acsnano.6c12908)
Supplement: Supplementary file 1 [file nn6c12908_si_001.pdf]

Supporting Information: Ion Transport Enhancement in Charge-Regulating Hydrogels

Pierre J. Walker,[*,†,§] David Beyer,[*,‡,¶,§] Christian Holm,[‡] and Zhen-Gang Wang[†]

†*Division of Chemistry and Chemical Engineering, California Institute of Technology, Pasadena, California, 91125, USA*

‡*Institute for Computational Physics, University of Stuttgart, D-70569 Stuttgart, Germany*

¶*Present Address: Department of Theoretical and Computational Biophysics, Max Planck Institute for Multidisciplinary Sciences, D-37077 Göttingen, Germany*

§*These authors contributed equally to this work.*

E-mail: pjwalker@caltech.edu; david.beyer@mpinat.mpg.de

1 Simulation Model and Method

1.1 Coarse-Grained Hydrogel Model

Our coarse-grained, implicit-solvent model for polyelectrolyte chains and ions is derived from the model of Kremer, Grest and Stevens.[S1,S2] This model represents monomers and ions as spherical, bead-like particles that interact via (1) repulsive steric interactions, (2) electrostatic interactions and (3) FENE bonds. Steric interactions between types of particles are modeled using the Weeks-Chandler-Andersen (WCA) potential.[S3] The WCA potential

between particle types i and j is given by

$$V_{\mathrm{WCA}}^{ij}(r) = \begin{cases} 4\epsilon\left(\left(\frac{\sigma_i+\sigma_j}{2r}\right)^{12} - \left(\frac{\sigma_i+\sigma_j}{2r}\right)^{6}\right) + \epsilon & \text{if } r \leq 2^{\frac{1}{6}}\frac{\sigma_i+\sigma_j}{2} \\ 0 & \text{if } r > 2^{\frac{1}{6}}\frac{\sigma_i+\sigma_j}{2}, \end{cases} \tag{1}$$

where $\epsilon = k_{\mathrm{B}}T$, with $T = 298.15\,\mathrm{K}$, is the energy scale of the interaction. σ_i is a measure for the bead diameter of particle type i which we set to $\sigma = 0.355\,\mathrm{nm}$ for all species except the cations X^+, which have a smaller diameter of $\sigma_{X^+} = \sigma/2 = 0.1775\,\mathrm{nm}$.

In addition to steric interactions, all charged particles also interact through the Coulomb potential[S4]

$$V_{\mathrm{Coulomb}}^{ij}(r) = \frac{\lambda_{\mathrm{B}} k_{\mathrm{B}} T z_i z_j}{r}, \tag{2}$$

where z_i is the charge number of particle type i. Here, the solvent of the system is taken to be water at $T = 298.15\,\mathrm{K}$ and is treated implicitly such that the Bjerrum length of the system is $\lambda_{\mathrm{B}} = 0.71\,\mathrm{nm}$. The Coulomb interactions in periodic boundary conditions are summed up using the P^3M method with a relative error of 10^{-4}.[S5–S7]

To connect monomers into polymer chains, we use FENE bonds,[S1] which are described by the interaction potential

$$V_{\mathrm{FENE}}(r) = \begin{cases} -\frac{k\Delta r_{\mathrm{max}}^2}{2}\ln\left(1 - \left(\frac{r}{\Delta r_{\mathrm{max}}}\right)^2\right) & \text{if } r \leq \Delta r_{\mathrm{max}} \\ \infty & \text{if } r > \Delta r_{\mathrm{max}}. \end{cases} \tag{3}$$

Throughout this work, we set the spring constant to $k = 30\,k_{\mathrm{B}}T/\sigma^2$ and use a value of $\Delta r_{\mathrm{max}} = 1.5\,\sigma$ for the maximum bond extension.

1.2 Hybrid MD/MC Scheme

We use a hybrid scheme combining Molecular Dynamics (MD) with Monte Carlo (MC) to simulate the molecular motions of our system as well as the acid-base reactions. At a fixed particle number, the positions of all particles (small ions and monomers) are propagated using Langevin dynamics with Gaussian white noise. In the simulations, we solve the Langevin equations for all particle positions numerically using the Velocity-Verlet integrator with a time step of $\Delta t = 0.01\,\tau$, where τ is the Lennard-Jones time scale. We set the bare friction coefficient to $\gamma = 1.0\,\tau^{-1}$, corresponding to a diffusion coefficient of $D = 1.0\,\sigma^2\tau^{-1}$ for non-interacting beads.

The monomers of our hydrogel are weakly acidic groups participating in the acid-base reaction

$$\mathrm{HA} \rightleftharpoons \mathrm{A}^- + \mathrm{H}^+, \tag{4}$$

which is governed by the equilibrium constant $K_\mathrm{a} = 10^{-\mathrm{p}K_\mathrm{a}}$. To model this reaction, we use the Reaction Ensemble Monte Carlo method[S8,S9] (RxMC). In this method, elementary reaction steps are represented by Monte Carlo moves that are probabilistically accepted. A single MC move proceeds as follows. First, the *extent of reaction* ξ is randomly selected ($\xi = +1$ for the forward reaction in Equation 4, $\xi = -1$ for the reverse reaction in Equation 4). Next reactant particles are randomly selected, i.e. a monomer HA if $\xi = +1$ or a monomer A^- and counterion H^+ if $\xi = -1$. Then, a new state is proposed by changing the identity of the selected monomer according to Equation 4 and either deleting the selected counterion H^+ (if $\xi = -1$) or inserting an additional counterion H^+ at a random position into the simulation box (if $\xi = +1$). Subsequently, the proposed new state (n) is accepted with a probability given by

$$P^{\mathrm{RxMC}}_{\mathrm{n,o}} = \min\left\{1, \frac{N^\mathrm{o}_\mathrm{HA}!N^\mathrm{o}_{\mathrm{A}^-}!N^\mathrm{o}_{\mathrm{H}^+}!(K_\mathrm{A} n^\ominus V)^\xi}{(N^\mathrm{o}_\mathrm{HA} - \xi)!\left(N^\mathrm{o}_{\mathrm{A}^-} + \xi\right)!\left(N^\mathrm{o}_{\mathrm{H}^+} + \xi\right)!} \exp\left(-\beta \Delta \mathcal{U}_{\mathrm{n,o}}\right)\right\}. \tag{5}$$

In this equation, N^o_i denotes the particle number of species i in the old state (o), $n^\ominus$ is the

number density corresponding to the reference concentration $c^{\ominus} = 1\,\mathrm{M}$, V is the volume of the simulation box and $\Delta\mathcal{U}_{\mathrm{n,o}}$ is the change in potential energy between the old state and the proposed new state. If the proposed new state is rejected, the old state is retained.

In the simulations, the MD integration steps are combined with MC moves by performing alternating cycles consisting of 10^3 MD and 10^3 MC steps respectively. To test the influence of the ratio of MD and MC steps, we also perform simulations where the number of MC steps per cycle is either reduced or enhanced by a factor of 10.

2 Supplementary Figures

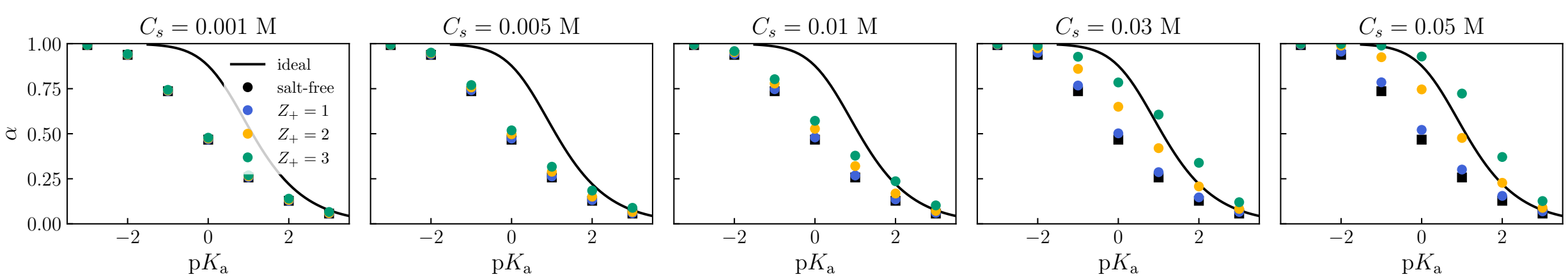

Figure S1: Variation in the hydrogel degree of ionization as a function of the pK_a value and for different salt concentrations and valencies. The black curves correspond to the theoretical prediction in the ideal limit.

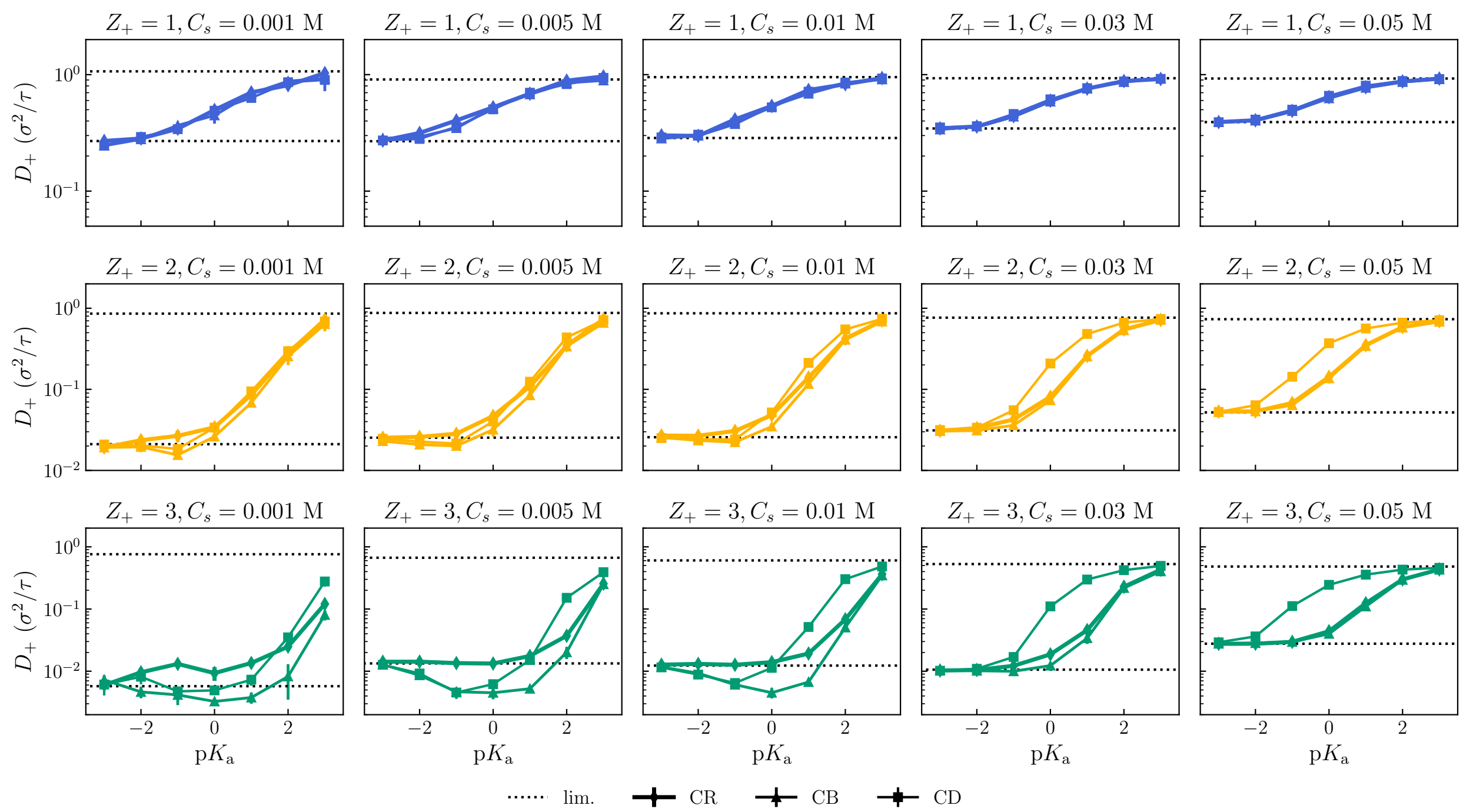

Figure S2: pK_a-dependence of diffusion coefficients obtained for mono-, di- and trivalent cations at different salt concentrations. The plots compare results obtained from simulations accounting for variations in the charge density (CD), competitive binding (CB) and dynamical charge regulation (CR). Dotted horizontal lines denote the diffusion coefficients obtained in the limits of fully ionized and neutral hydrogels.

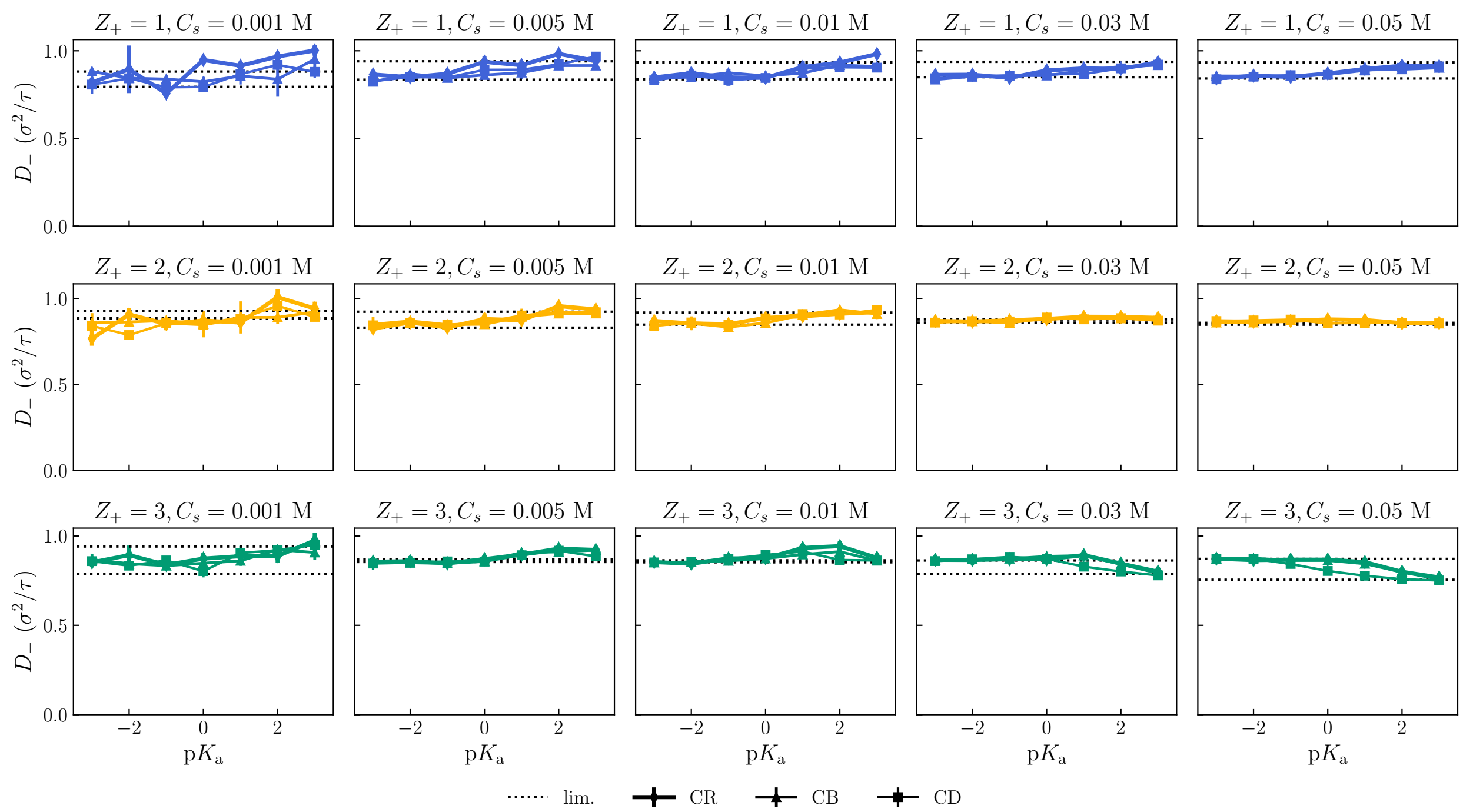

Figure S3: pK_a-dependence of monovalent anion diffusion coefficients obtained in the presence of mono-, di- and trivalent cations at different salt concentrations. The plots compare results obtained from simulations accounting for variations in the charge density (CD), competitive binding (CB) and dynamical charge regulation (CR). Dotted horizontal lines denote the diffusion coefficients obtained in the limits of fully ionized and neutral hydrogels.

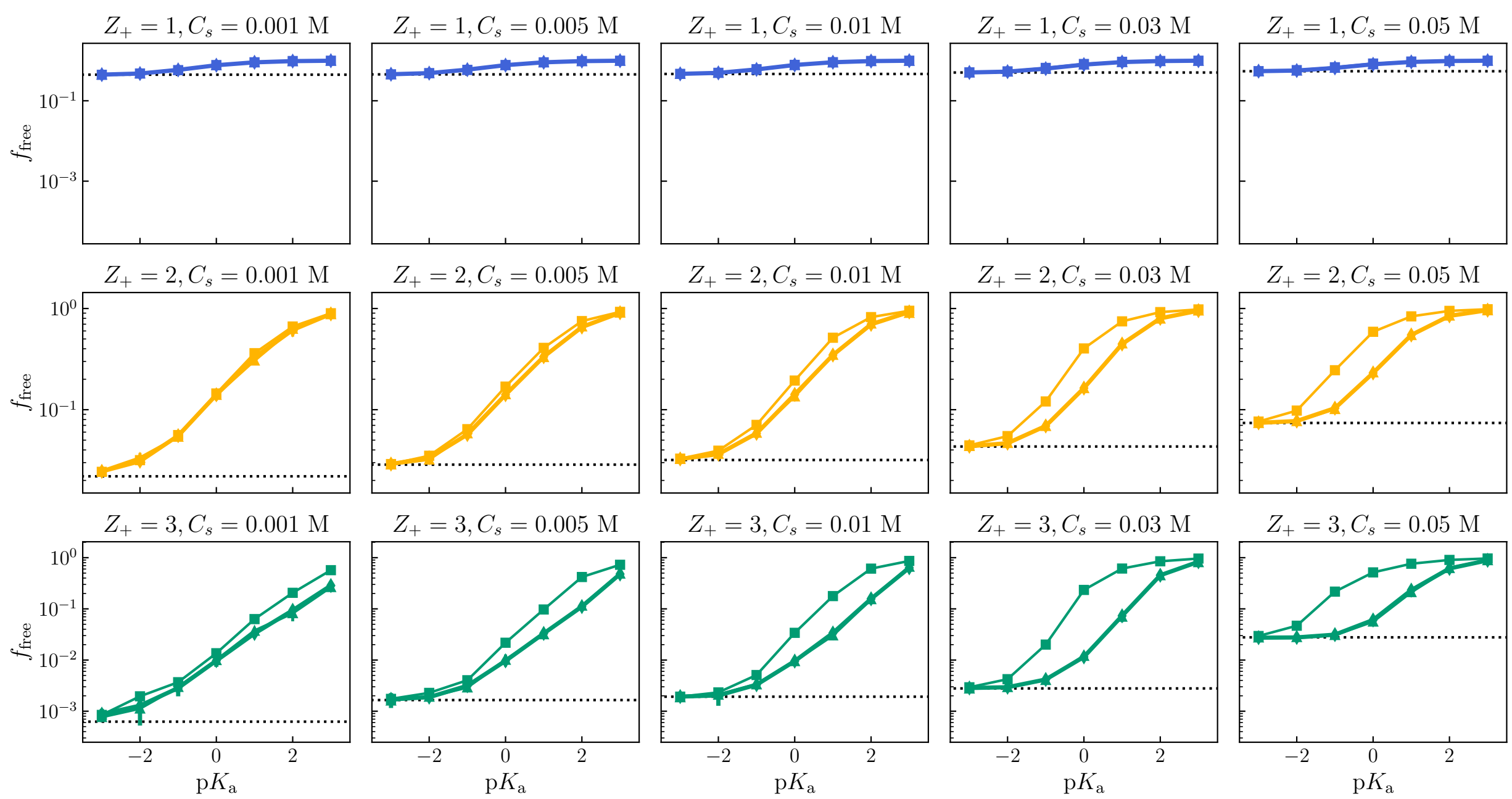

Figure S4: pK_{a}-dependence of the fraction of unbound cations obtained for mono-, di- and trivalent cations at different salt concentrations. The plots compare results obtained from simulations accounting for variations in the charge density (CD), competitive binding (CB) and dynamical charge regulation (CR). Dotted horizontal lines correspond to the fraction of unbound cations obtained in the limit of a fully ionized hydrogel.

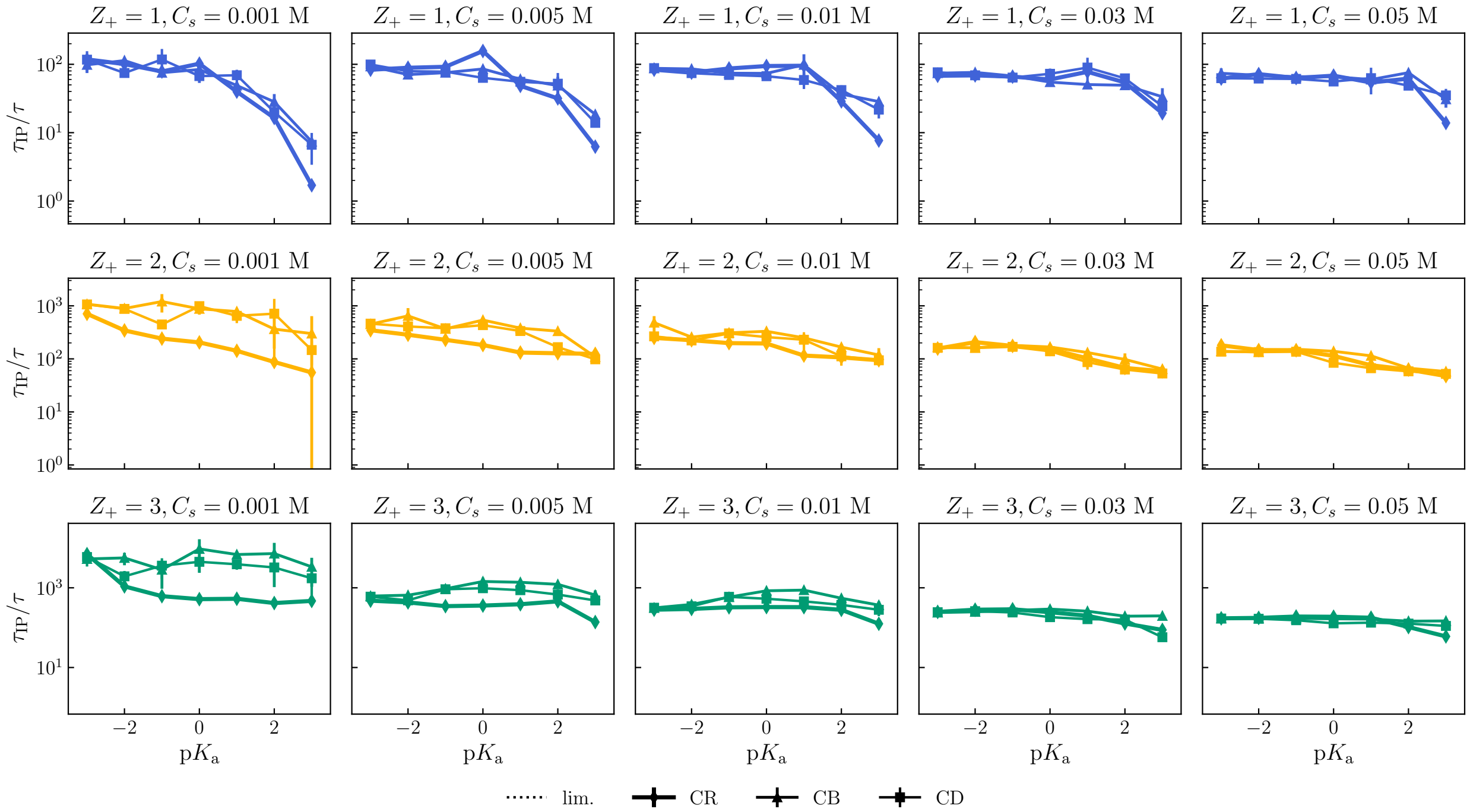

Figure S5: pK_{a}-dependence of the ion pair relaxation time obtained in the presence of mono-, di- and trivalent salts at different concentrations. The plots compare results obtained from simulations accounting for variations in the charge density (CD), competitive binding (CB) and dynamical charge regulation (CR).

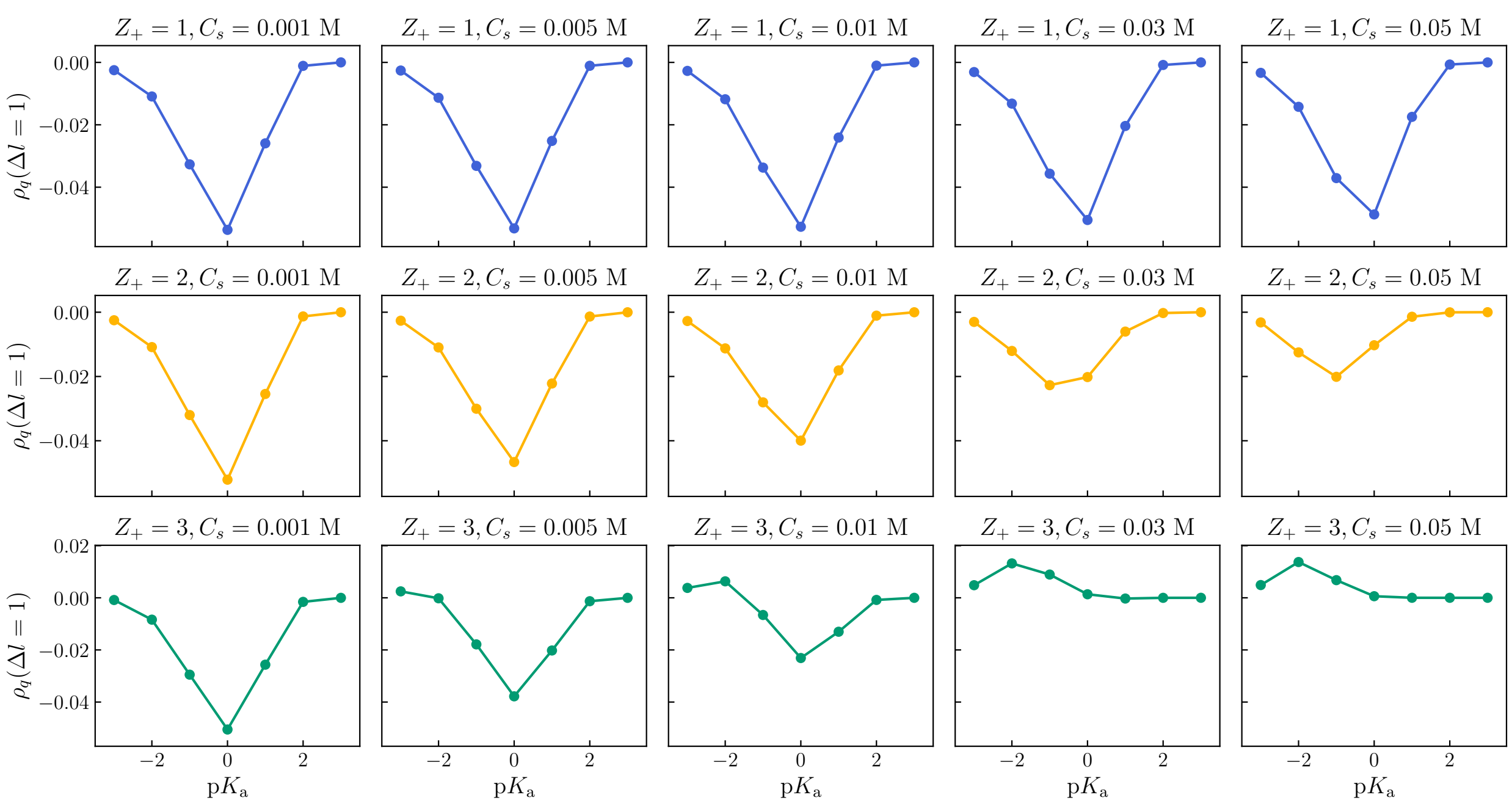

Figure S6: Nearest-neighbor polymer backbone charge autocorrelation function $\rho_q(\Delta l = 1)$ as a function of pK_{a} and obtained for different salt valencies and concentrations.

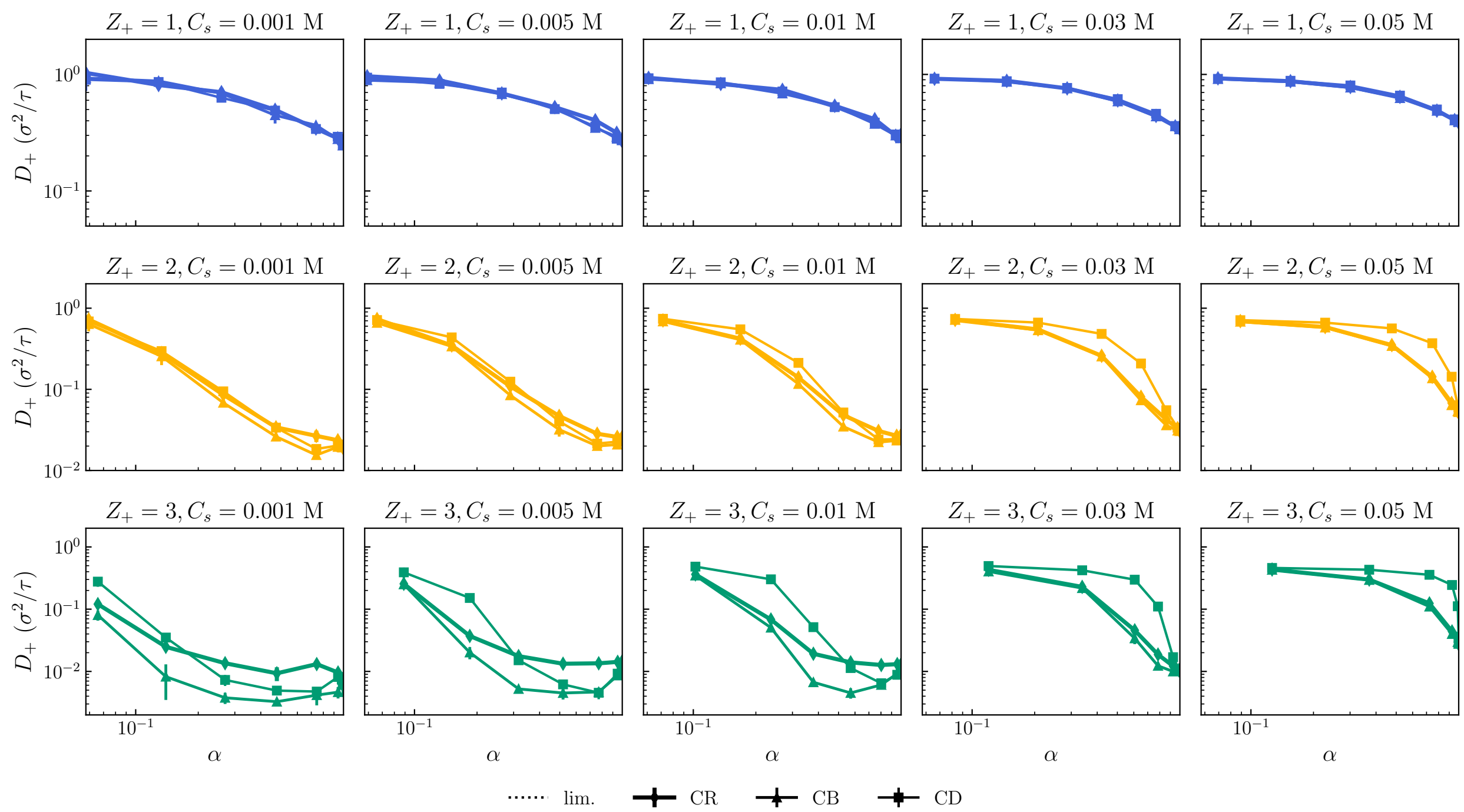

Figure S7: Diffusion coefficients obtained for mono-, di- and trivalent cations at different salt concentrations, plotted as a function of the hydrogel backbone degree of ionization α. The plots compare results obtained from simulations accounting for variations in the charge density (CD), competitive binding (CB) and dynamical charge regulation (CR).

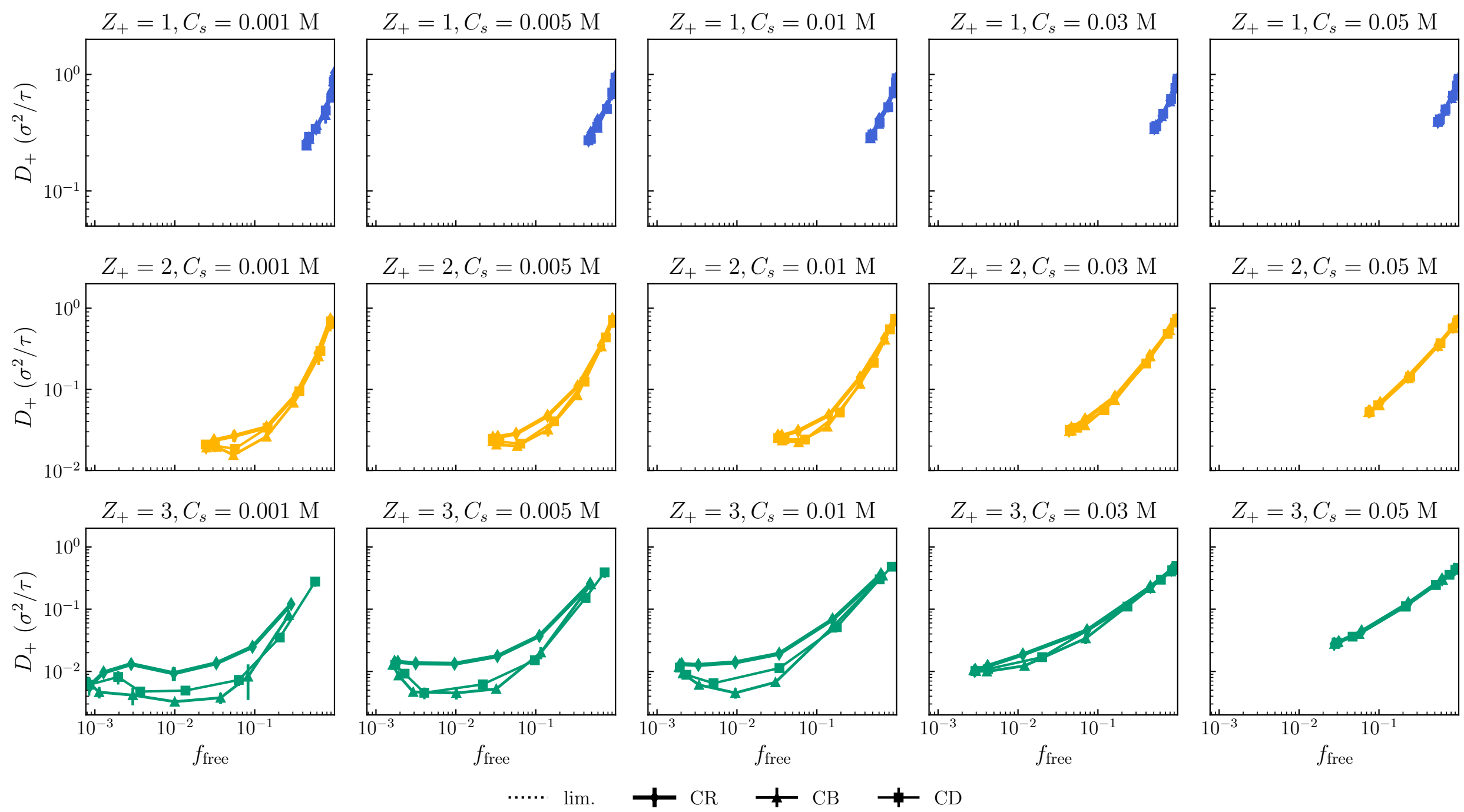

Figure S8: Diffusion coefficients obtained for mono-, di- and trivalent cations at different salt concentrations, plotted as a function of the fraction of unbound cations. The plots compare results obtained from simulations accounting for variations in the charge density (CD), competitive binding (CB) and dynamical charge regulation (CR).

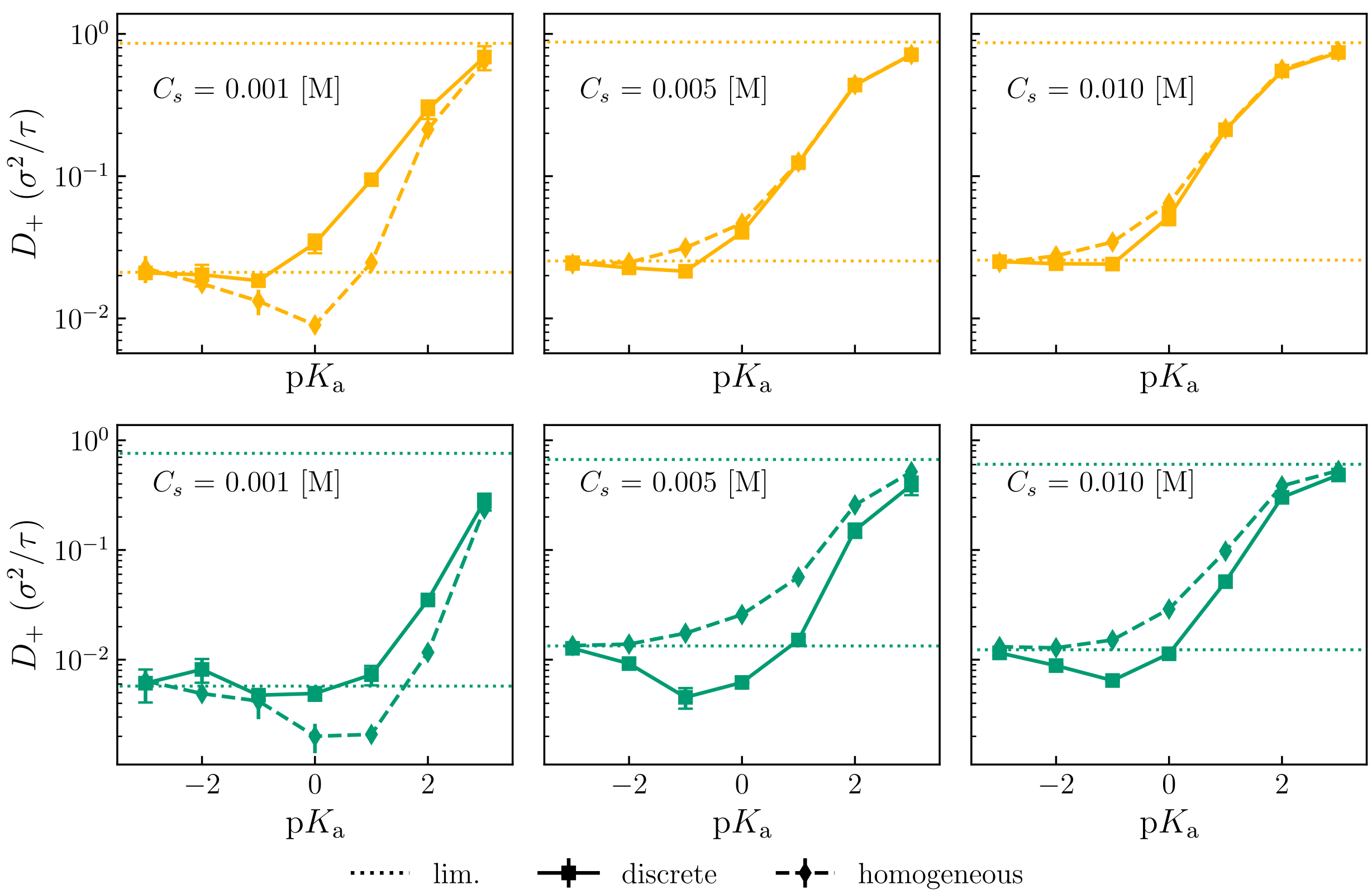

Figure S9: Diffusion coefficients obtained for divalent cations (upper row) and trivalent cations (lower row) at different salt concentrations, plotted as a function of the pK_a value. The plots compare results obtained from charge density simulations utilizing charge profiles obtained from prior RxMC simulations ("discrete") and profiles for which the same charge was distributed homogeneously across all monomers comprising hydrogel network ("homogeneous"). Dotted horizontal lines denote the diffusion coefficients obtained in the limits of fully ionized and neutral hydrogels.

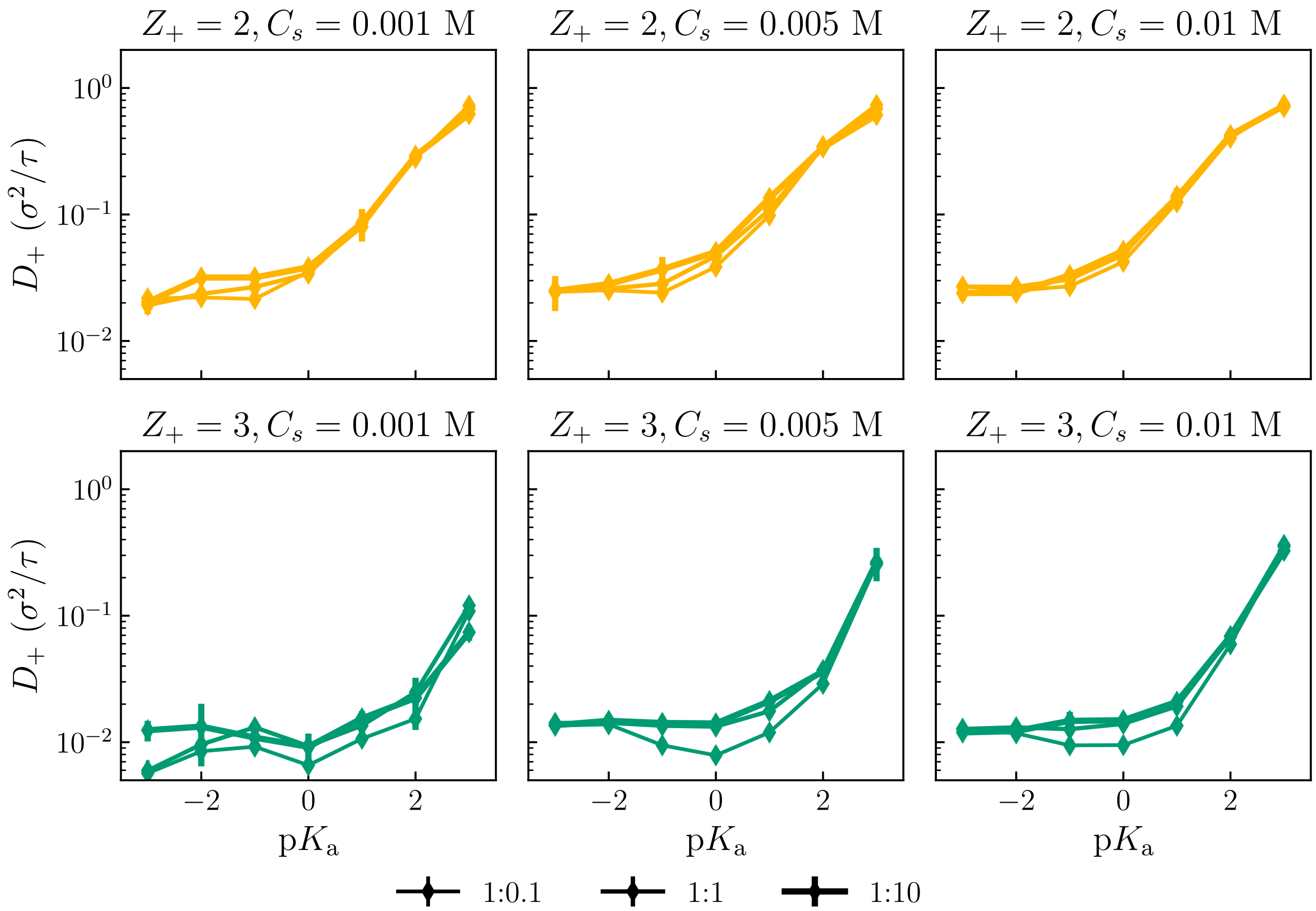

Figure S10: Diffusion coefficients obtained for divalent cations (upper row) and trivalent cations (lower row) at different salt concentrations, plotted as a function of the pK_a value. The plots compare results obtained from varying the ratio of MD to MC steps within the RxMC + MD simulations.

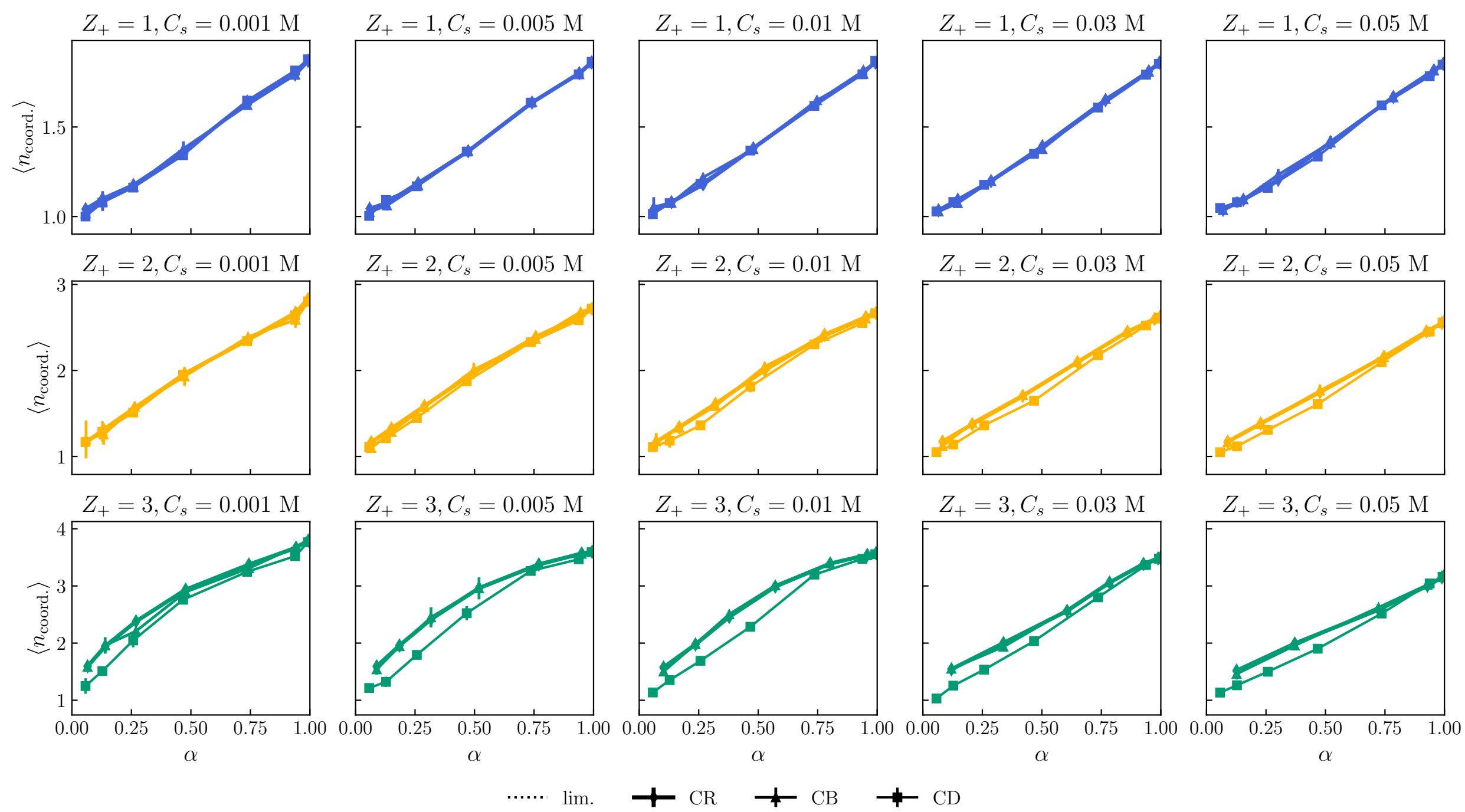

Figure S11: pK_a-dependence of the coordination number of each cation, i.e., the mean number of monomers electrostatically bound to a cation, obtained for mono-, di- and trivalent cations at different salt concentrations. The plots compare results obtained from simulations accounting for variations in the charge density (CD), competitive binding (CB) and dynamical charge regulation (CR). Dotted horizontal lines correspond to the fraction of unbound cations obtained in the limit of a fully ionized hydrogel.

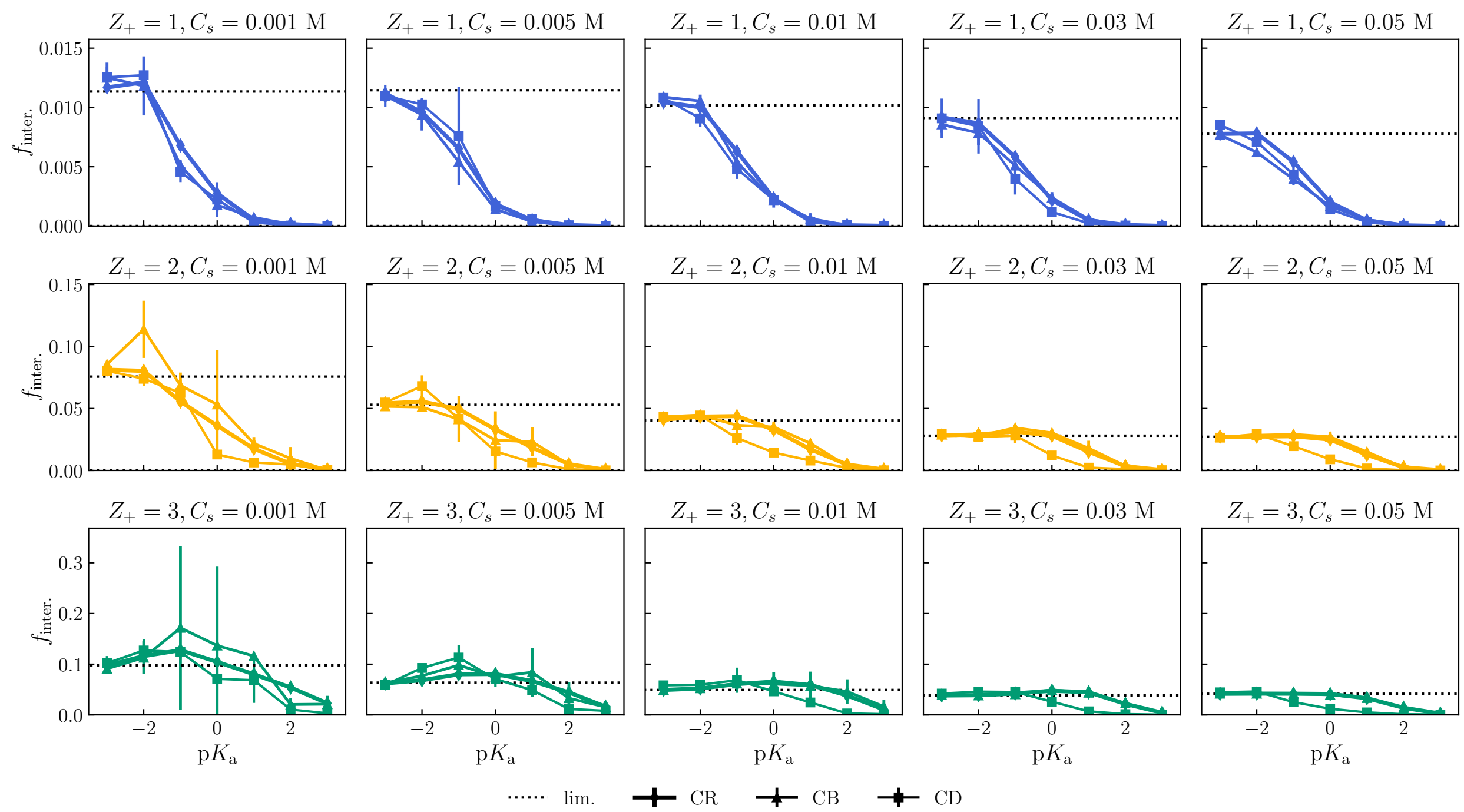

Figure S12: pK_a-dependence of the fraction of cations physicall crosslinking different strands of the hydrogel, obtained for mono-, di- and trivalent cations at different salt concentrations. The plots compare results obtained from simulations accounting for variations in the charge density (CD), competitive binding (CB) and dynamical charge regulation (CR). Dotted horizontal lines correspond to the fraction of unbound cations obtained in the limit of a fully ionized hydrogel.

References

(S1) Kremer, K.; Grest, G. S. Dynamics of Entangled Linear Polymer Melts: A Molecular-dynamics Simulation. *J. Chem. Phys.* **1990**, *92*, 5057–5086.

(S2) Stevens, M. J.; Kremer, K. Structure of Salt-Free Linear Polyelectrolytes. *Physical Review Letters* **1993**, *71*, 2228–2231.

(S3) Weeks, J. D.; Chandler, D.; Andersen, H. C. Role of Repulsive Forces in Determining the Equilibrium Structure of Simple Liquids. *The Journal of Chemical Physics* **1971**, *54*, 5237–5247.

(S4) Coulomb, C. A. C. A.; Fossier, i.; Le Gouaz, Y.-M.; Adam, e.; Burndy Library, d. D.; Académie royale des sciences (France) *Mémoires Sur l'électricité et La Magnétisme*; Paris : Chez Bachelier, libraire ..., 1789.

(S5) Hockney, R. W.; Eastwood, J. W. *Computer Simulation Using Particles*; CRC Press: Boca Raton, 1988.

(S6) Deserno, M.; Holm, C. How to Mesh up Ewald Sums. I. A Theoretical and Numerical Comparison of Various Particle Mesh Routines. *The Journal of Chemical Physics* **1998**, *109*, 7678–7693.

(S7) Deserno, M.; Holm, C. How to Mesh up Ewald Sums. II. An Accurate Error Estimate for the Particle–Particle–Particle-Mesh Algorithm. *The Journal of Chemical Physics* **1998**, *109*, 7694–7701.

(S8) Johnson, J. K.; Panagiotopoulos, A. Z.; Gubbins, K. E. Reactive Canonical Monte Carlo: A New Simulation Technique for Reacting or Associating Fluids. *Mol. Phys.* **1994**, *81*, 717–733.

(S9) Smith, W. R.; Triska, B. The Reaction Ensemble Method for the Computer Simulation

of Chemical and Phase Equilibria. I. Theory and Basic Examples. *J. Chem. Phys.* **1994**, *100*, 3019–3027.
